# Structural Diversity and Biological Activities of Cyclic Depsipeptides from Fungi

**DOI:** 10.3390/molecules23010169

**Published:** 2018-01-15

**Authors:** Xiaohan Wang, Xiao Gong, Peng Li, Daowan Lai, Ligang Zhou

**Affiliations:** Department of Plant Pathology, College of Plant Protection, China Agricultural University, Beijing 100193, China; wangxiaohan99@126.com (X.W.); gongxiao1994@163.com (X.G.); ytmuyue1989@163.com (P.L.); dwlai@cau.edu.cn (D.L.)

**Keywords:** cyclodepsipeptides, fungi, biological activities, occurrence, applications

## Abstract

Cyclic depsipeptides (CDPs) are cyclopeptides in which amide groups are replaced by corresponding lactone bonds due to the presence of a hydroxylated carboxylic acid in the peptide structure. These peptides sometimes display additional chemical modifications, including unusual amino acid residues in their structures. This review highlights the occurrence, structures and biological activities of the fungal CDPs reported until October 2017. About 352 fungal CDPs belonging to the groups of cyclic tri-, tetra-, penta-, hexa-, hepta-, octa-, nona-, deca-, and tridecadepsipeptides have been isolated from fungi. These metabolites are mainly reported from the genera *Acremonium*, *Alternaria*, *Aspergillus*, *Beauveria*, *Fusarium*, *Isaria*, *Metarhizium*, *Penicillium*, and *Rosellina*. They are known to exhibit various biological activities such as cytotoxic, phytotoxic, antimicrobial, antiviral, anthelmintic, insecticidal, antimalarial, antitumoral and enzyme-inhibitory activities. Some CDPs (i.e., PF1022A, enniatins and destruxins) have been applied as pharmaceuticals and agrochemicals.

## 1. Introduction

Cyclic depsipeptides (CDPs), also known as cyclodepsipeptides or peptolides, are cyclooligomers in which one or more amino acid is replaced by a hydroxylated carboxylic acid, resulting in the formation of at least one lactone bond in the core ring. They are biosynthesized by non-ribosomal peptide synthetases (NRPS) in combination with either polyketide synthase (PKS) or fatty acid (FA) synthase enzyme systems [1,2,3]. CDPs are widely distributed in bacteria [4], fungi [1], plants [5,6], algae [7], sponges [8], and other marine organisms [9,10,11,12,13]. Here, we focus on fungal CDPs which include cyclic tri-, tetra-, penta-, hexa-, hepta-, octa-, nona-, deca-, and tridecadepsipeptides though fungi can produce large amounts of cyclic peptides without any lactone bond in the core ring [14,15]. Some fungal CDPs such as beauvericins, destruxins, enniatins have been well characterized [16,17,18,19]. Special reviews covering chemical synthesis [16], biosynthesis [20], chemical classification [3], as well as applications [21,22] of fungal CDPs are also available. In this review, we describe the occurrence, biological activities, and structures of all hitherto reported fungal CDPs to assess which of them merit further study for purposes of drug development as well as for clarification of their physiological and ecological functions. We still classify fungal CDPs based on the total amounts of amino and hydroxylated carboxylic acids though a review about the classification of CDPs based on the hydroxylated carboxylic acid(s) involved in the ring lacone has just been published [3].

## 2. Cyclic Tridepsipeptides

**Figure 1:**
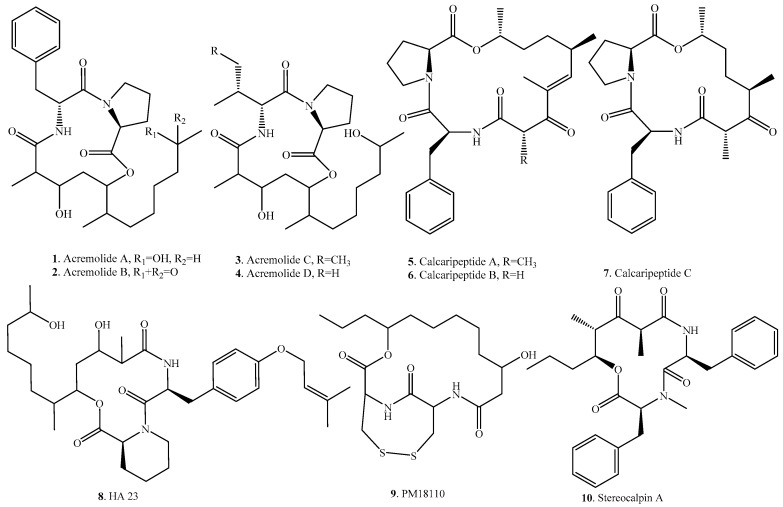
Structures of the cyclic tridepsipeptides isolated from fungi.

Cyclic tridepsipeptides usually contain two amino acids and one hydroxylated carboxylic acid. They were found in the genera *Acremonium*, *Calcarisporium*, *Fusarium*, *Phomopsis* and *Ramalina*. The occurrence and biological activities of fungal cyclic tridepsipeptides are listed in Table 1, and their structures are shown in Figure 1.

Ten cyclic tridepsipeptides have been isolated from fungi so far. Acremolides A–D (**1**–**4**) were isolated from an Australian marine-derived *Acremonium* sp. MST-MF588a obtained from a sediment sample [23]. Calcaripeptides A (**5**), B (**6**), and C (**7**) were identified from *Calcarisporium* sp. strain KF525, which was isolated from German Wadden Sea [24]. HA23 (**8**), a cyclic tridepsipeptide of mixed peptide-polyketide origins, was isolated from *Fusarium* sp. CANU-HA23 [25].

PM181110 (**9**) was identified from the endophytic fungus *Phomopsis glabrae* isolated from the leaves of *Pongamia pinnata*, and exhibited anticancer activity against 40 human cancer cell lines with a mean IC_50_ value of 0.089 μM. The structure of this compound has a disulfide ring, which possibly contributed to the biological activity [26].

Stereocalpin A (**10**) was isolated from the endophytic fungus *Ramalina terebrata* associated with the Antarctic lichen *Stereocaulon alpinum*. This CDP is unique in that its structure contains a 5-hydroxy-2,4-dimethyl-3-oxo-octanoic acid. It showed moderate cytotoxic activity against three human solid tumor cell lines (i.e., colon carcinoma cell line HT-29, skin carcinoma cell line B16/F10, and liver carcinoma cell line HepG2), and weak inhibitory activity against protein tyrosine phosphatase 1B (PTP1B) [27]. Further investigation of the mechanism showed that stereocalpin A (**10**) inhibited the expression of adhesion molecules in activated muscle cells. These results suggest that this compound has the potential to exert a protective effect by modulating inflammation within the atherosclerotic lesion [28].

## 3. Cyclic Tetradepsipeptides

**Figure 2:**
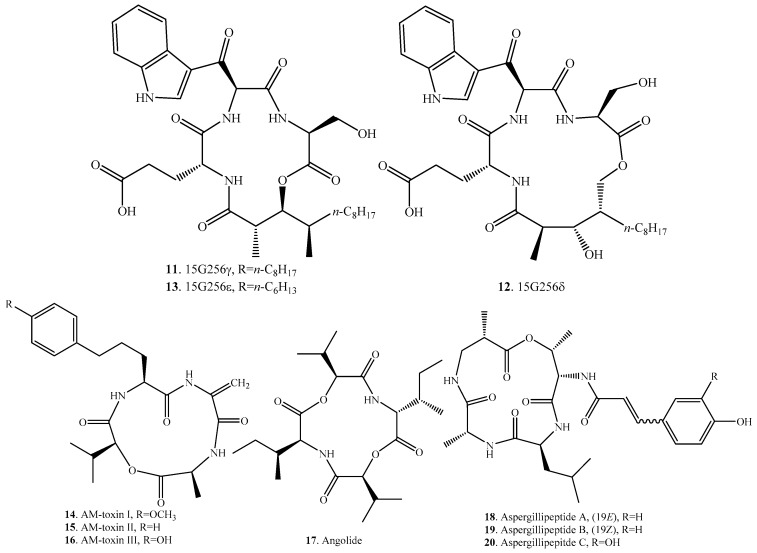

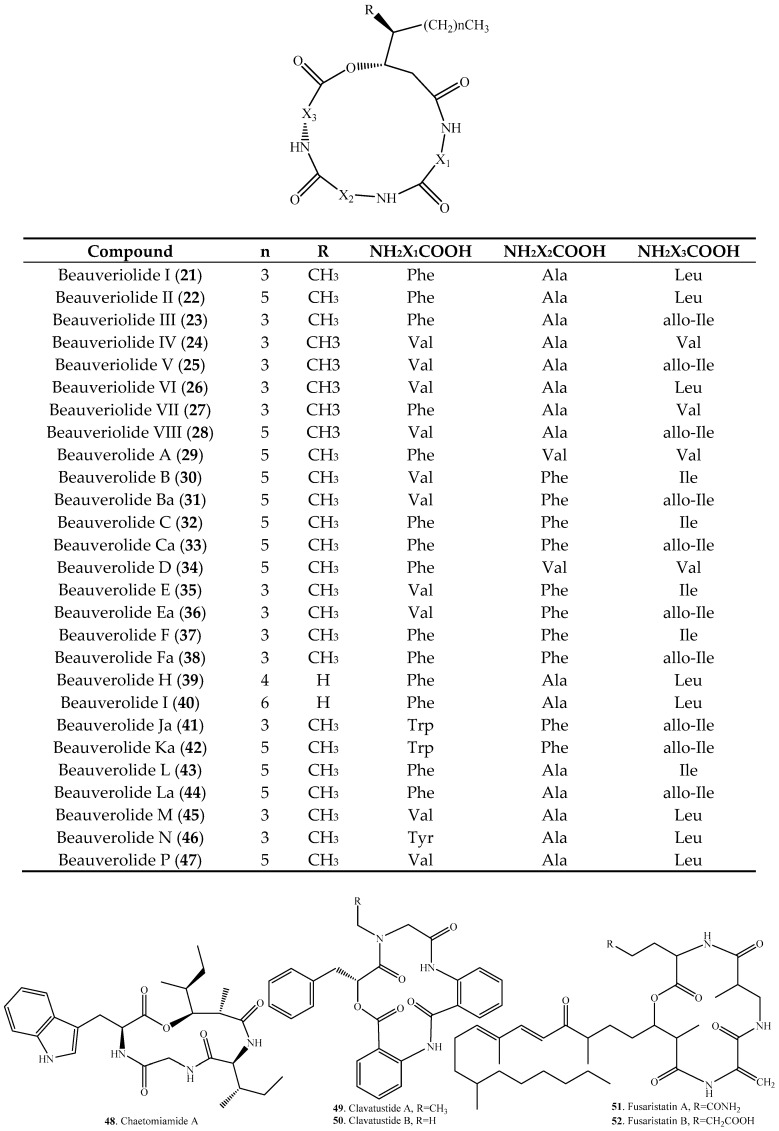

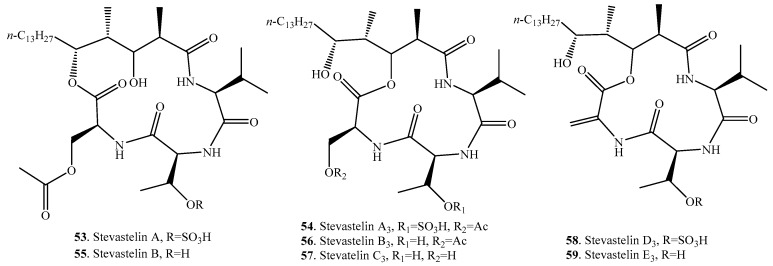
Structures of the cyclic tetradepsipeptides isolated from fungi.

Forty nine cyclic tetradepsipeptides have been isolated from fungi so far. They have been found mainly in the genera *Alternaria*, *Aspergillus*, *Beauveria*, *Fusarium*, *Hypoxylon*, and *Penicillium*. Their occurrences in fungi, and biological activities are listed in Table 2, and the structures are provided in Figure 2.

15G256γ (**11**), δ (**12**) and ε (**13**) were isolated from the marine fungus *Hypoxylon oceanicum* (LL-15G256) [29,30]. They showed moderate antifungal activity against the plant pathogenic fungi in greenhouse tests and human fungal pathogens in vitro. Microscopic examination of treated fungi suggested that the compounds displayed inhibition on cell wall biosynthesis [31].

AM-toxins I (**14**), II (**15**) and III (**16**), which were host-specific phytotoxins, were isolated from *Alternaria alternata* apple pathotype [32,33,34].

Aspergillipeptides A (**18**), B (**19**), and C (**20**) were obtained from *Aspergillus* sp. SCSGAF 41501 from China South Sea gorgonian *Melitodes squamata*. Aspergillipeptide C (**20**) showed strong antifouling activity against *Bugula neritina* larvae settlement [35].

Beauveriolides I-VIII (**21**–**28**) were isolated from *Beauveria* sp. [36,37,38]. Among them, beauveriolide I (**21**) displayed insecticidal activity on *Spodoptera litura* and *Callosobruchus chinensis* [36]. Beauveriolide III (**23**) selectively inhibited sterol *O*-acyltransferase 1 (SOAT1) in a cell-based assay [39].

Clavatustides A (**49**) and B (**50**) were identified from the cultured mycelia and broth of *Aspergillus clavatus* C2WU. The fungus was isolated from the crab *Xenograpsus testudinatus*, which lived at extreme, toxic habitat around the sulphur-rich hydrothermal vents in Taiwan Kueishantao. Both compounds suppressed the proliferation of hepatocellular carcinoma (HCC) cell lines (HepG2, SMMC-7721 and Bel-7402), and induced an accumulation of HepG2 cells in G1 phase and reduction of cells in S phase [40]. CCNE2 (cyclin E2) was proved to be the key regulator of clavatustide B-induced G1-S transition blocking in several cancer cell lines by using real-time PCR [41].

Fusaristatins A (**51**) and B (**52**) were identified in the endophytic fungus *Fusarium* sp. YG-45. Both compounds showed a moderate inhibitory effect on topoisomerases I and II. They also showed the growth-inhibitory activity toward lung cancer cells LU 65 [42]. Fusaristatin A (**51**) also displayed an inhibitory effect on the fungus *Glomerella acutata* [43].

A series of stevastelins were obtained from *Penicillium* sp. NK374186 which was isolated from the soil collected in Niigata of Japan [44,45,46]. They inhibited interleukin-2 or interleukin-6 dependent gene expression but did not inhibit the phosphatase activity of calcineurin. Stevastelins were considered as the potential immunosuppressants [47].

## 4. Cyclic Pentadepsipeptides

**Figure 3:**
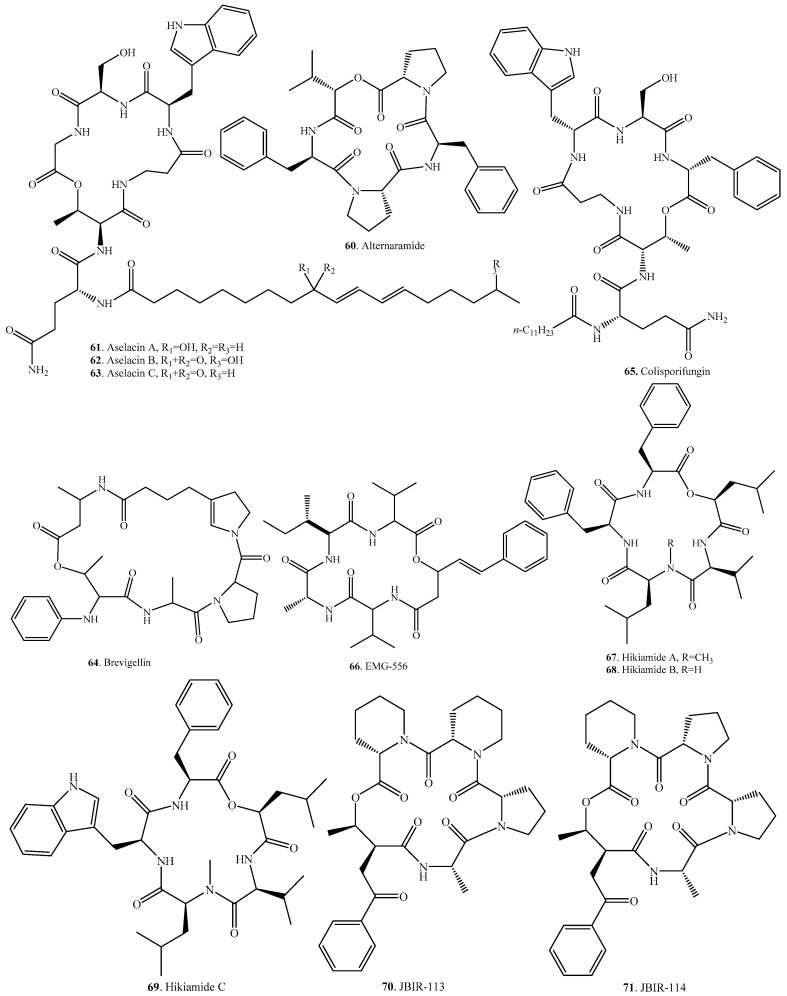

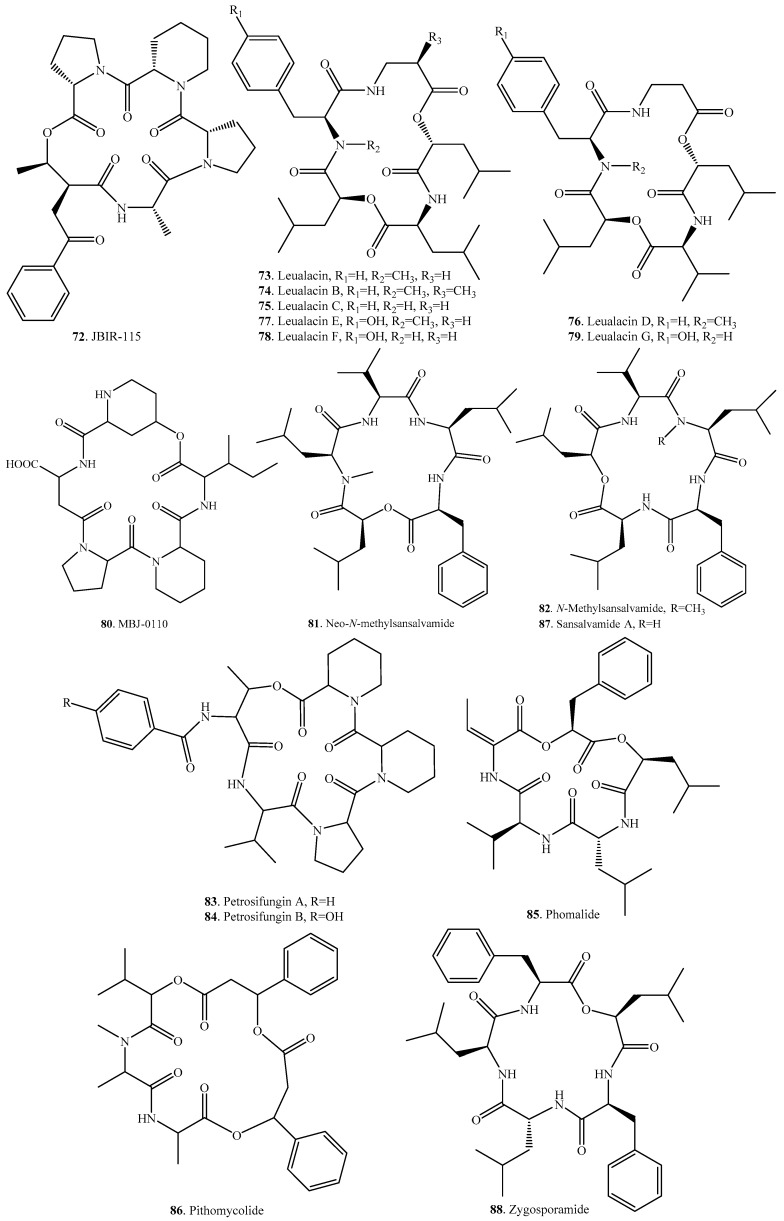
Structures of the cyclic pentadepsipeptides isolated from fungi.

Cyclic pentadepsipeptides have been isolated from the genera *Acremonium*, *Alternaria*, *Fusarium*, *Hapsidospora*, and *Penicillium*. Their occurrences and biological activities are listed in Table 3, and their structures are provided in Figure 3.

Alternaramide (**60**) was identified in the marine-derived fungus *Alternaria* sp. SF-5016, and showed weak antibiotic activity on *Bacillus subtilis* and *Staphylococcus aureus* [58]. This compound also had inhibitory effects on inflammatory mediator expression through TLR4-MyD88-mediated inhibition of NF-κB and MAPK pathway signaling in lipopolysaccharide-stimulated RAW264.7 and BV2 cells [59].

Aselacins A (**61**), B (**62**) and C (**63**) were obtained in *Acremonium* spp. from the soil samples collected in Asela (Ethiopia). They had inhibitory activity on the binding of endothelin to its receptor. Among them, aselacin A (**61**) inhibited binding to receptors in both atrial and cerebral membranes with IC_50_ values of 20 μg/mL, approximately [60,61].

By means of epigenetic manipulation of the fungal metabolome, EGM-556 (**66**) was identified by addition of histone deacetylase inhibitor suberoylanilide hydroxamic acid into the culture of the Floridian marine sediment-derived fungus *Microascus* sp. [62].

Hikiamides A (**67**), B (**68**) and C (**69**) were obtained from *Fusairum* sp. TAMA 456 from a rotten wood sample collected in Hiki county of Japan, and induced adipocyte differentiation and mRNA expression of adiponectin in murine ST-3 preadipocyte cells [63].

JBIR-113 (**70**), JBIR-114 (**71**), and JBIR-115 (**72**) were identified in the marine-derived *Penicillium* sp. fS36 from an unidentified sponge collected near Takarajima Island of Japan [64]. Copper and manganese cations induced production of JBIR-113 (**70**), JBIR-114 (**71**), and JBIR-115 (**72**) in the endophytic fungus *Penicillium brasilianum* from *Melia azedarach*. JBIR-113 (**70**) exhibited weak antiparasitary acitivity against *Leishmania amazonensis* [65].

Leualacin (**73**) was first isolated from *Hapsidospora irregularis*. This compound inhibited the binding of *H*-nitrendipine to porcine heart membranes in vitro and lowered the blood pressure of spontaneous hypertensive rats to show its potential application as the calcium channel blocker for treatment of hypertension, angina, myocardial infarction, and arrhythmia [66,67]. Afterwards, six other anlogues, leualacins B–G (**74**–**79**) were obtained from this fungal species. Leualacin F (**78**) elicited the calcium influx in primary human lobar bronchial epithelial cells involving the TRPA1 channel [68].

Phomalide (**85**) was isolated from the pathogen *Phoma lingam* (teleomorph: *Leptosphaeria maculans*) of the blackleg disease of brassica crops. This compound showed host-selective phytotoxicity [69,70].

Sansalvamide A (**87**) was isolated from a marine fungus *Fusarium* sp. [71]. This compound possessed marked antitumor activity against 60 cancer cell lines such as human prostate cancer PC3, human breast cancer MDA-MB-231, and human melanoma WM-115 by inhibiting topoisomerase I [72]. *N*-Methylation of sansalvamide A (**87**) enhanced its antitumor potency and selectivity [73]. Its derivative H-10 exhibited antiproliferative effects against murine melanoma B16 cells and induced cell apoptosis [74]. Zygosporamide (**88**) was isolated from the marine-derived fungus *Zygosporium masonii*. This compound illustrated significant cytotoxic activity against SF-268 and RXF 393 cell lines [75].

## 5. Cyclic Hexadepsipeptides

**Figure 4:**
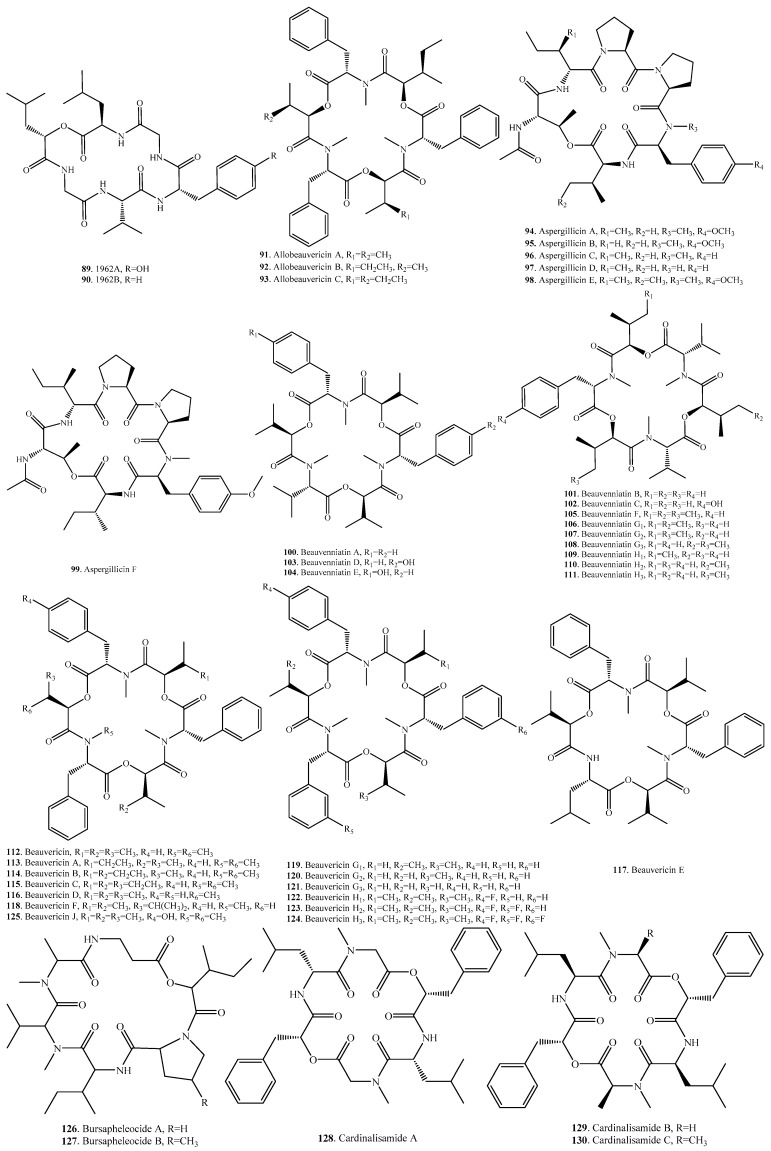

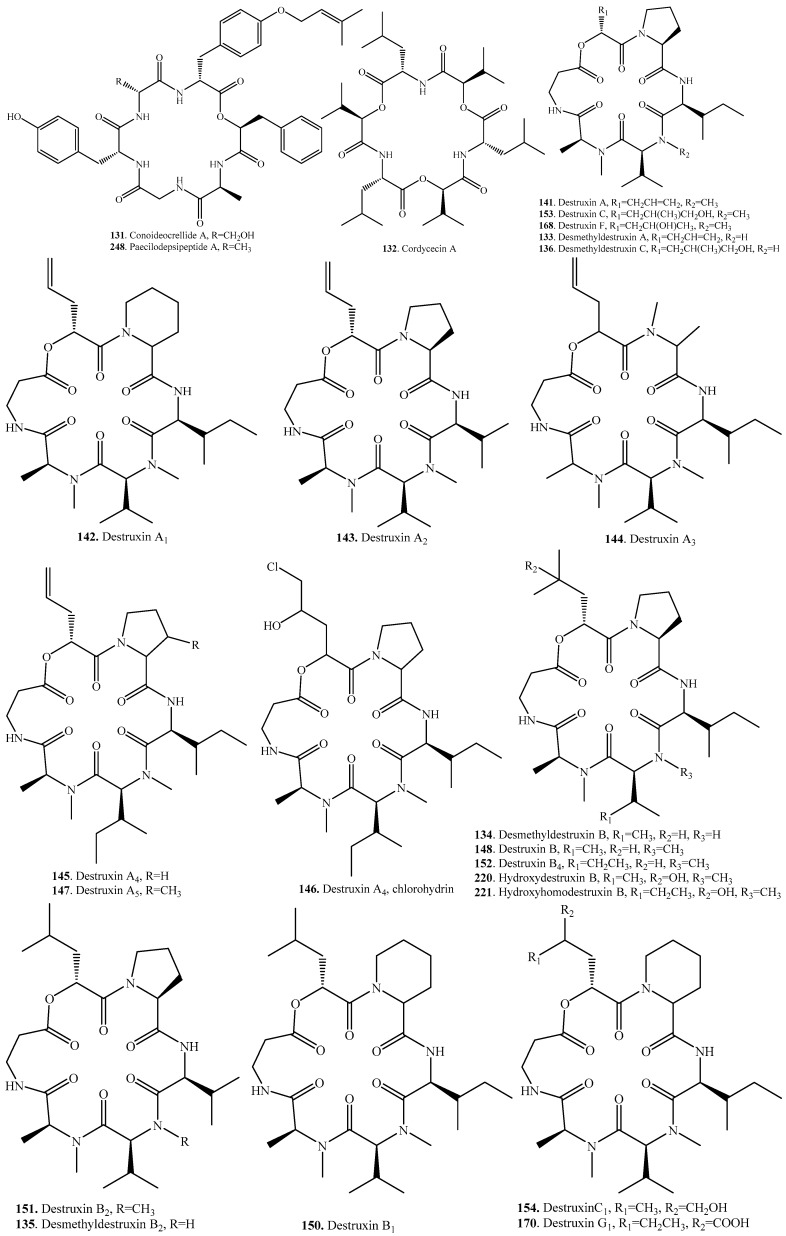

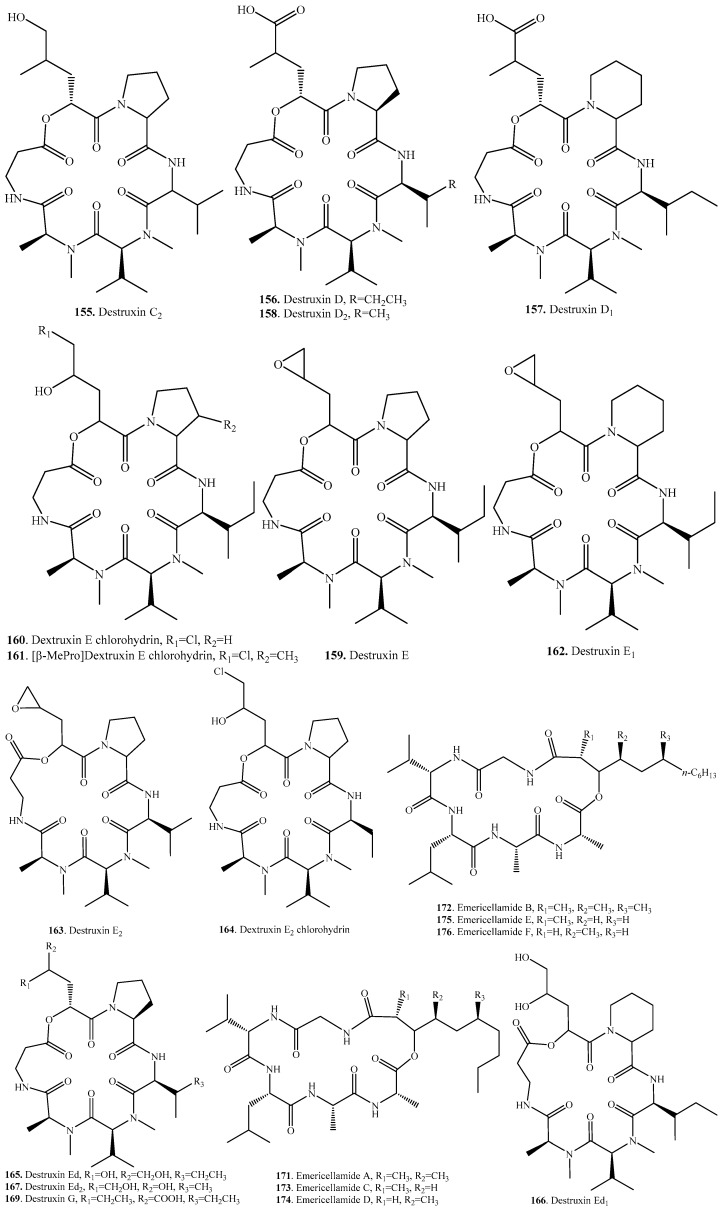

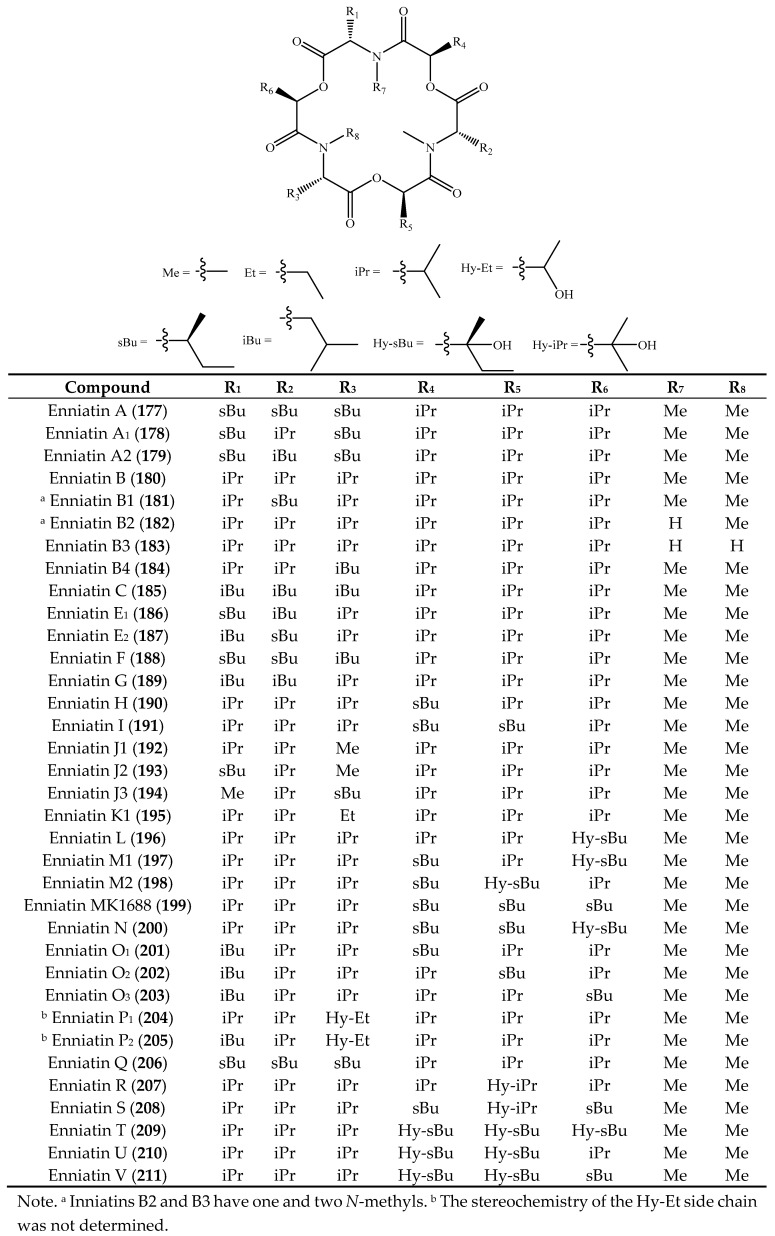

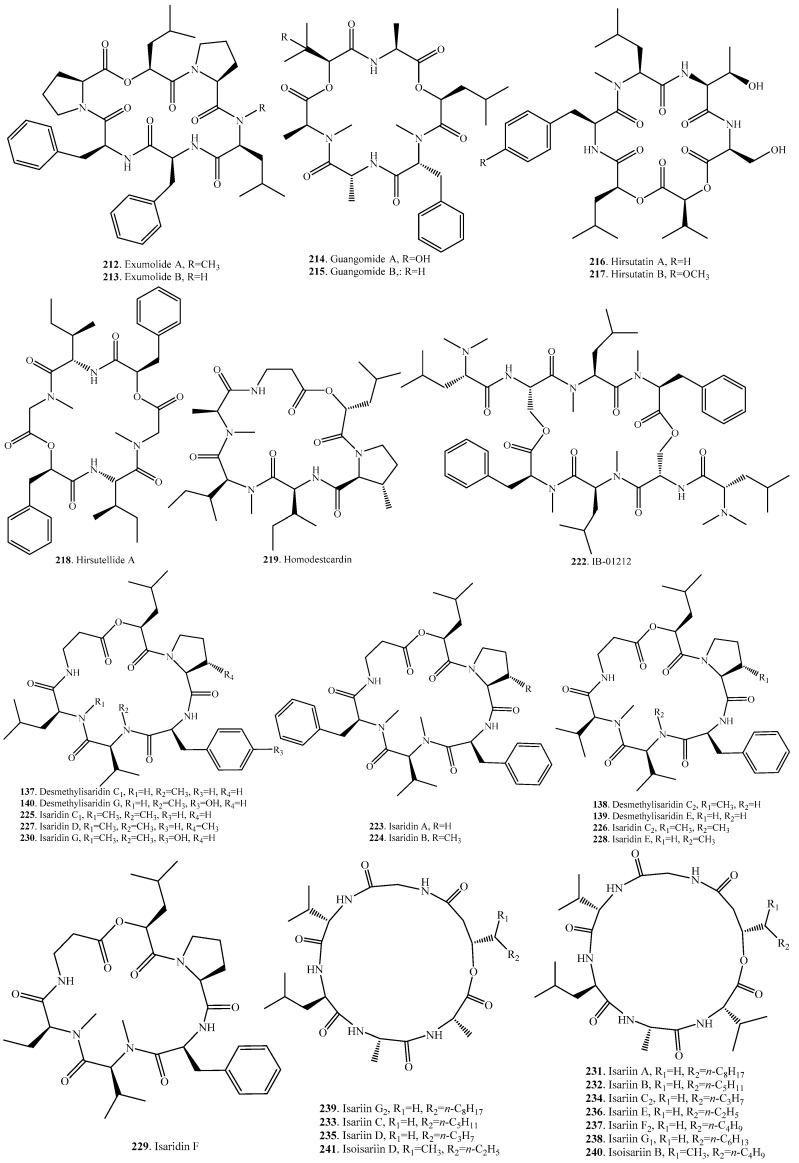

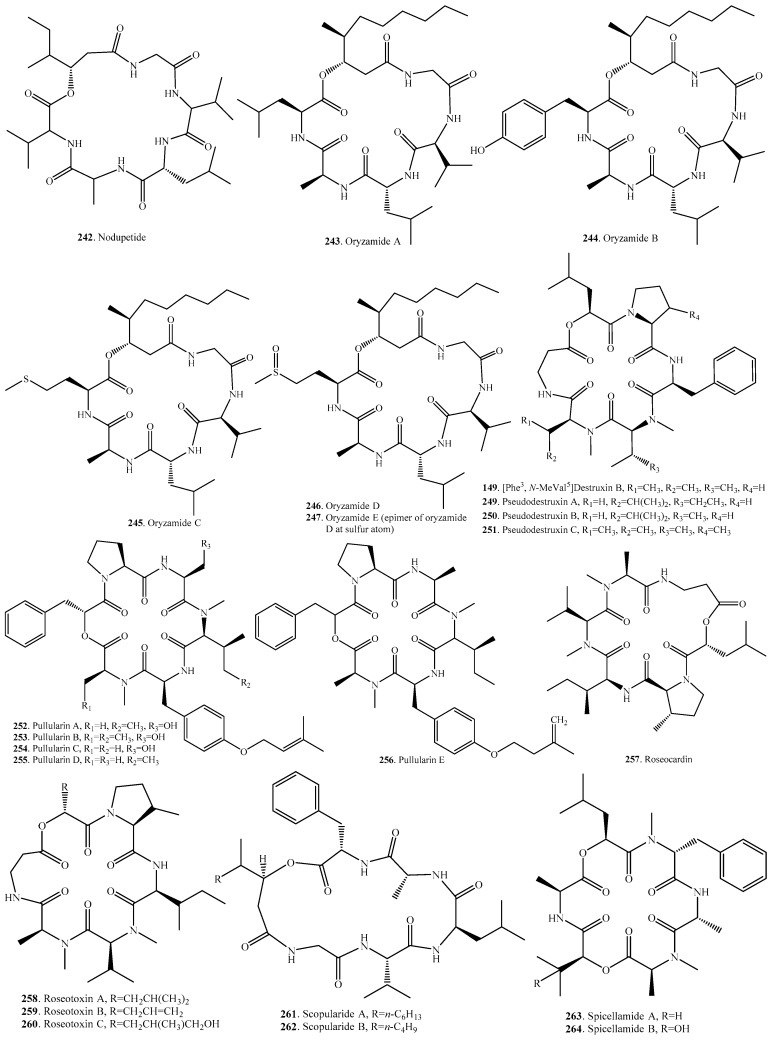

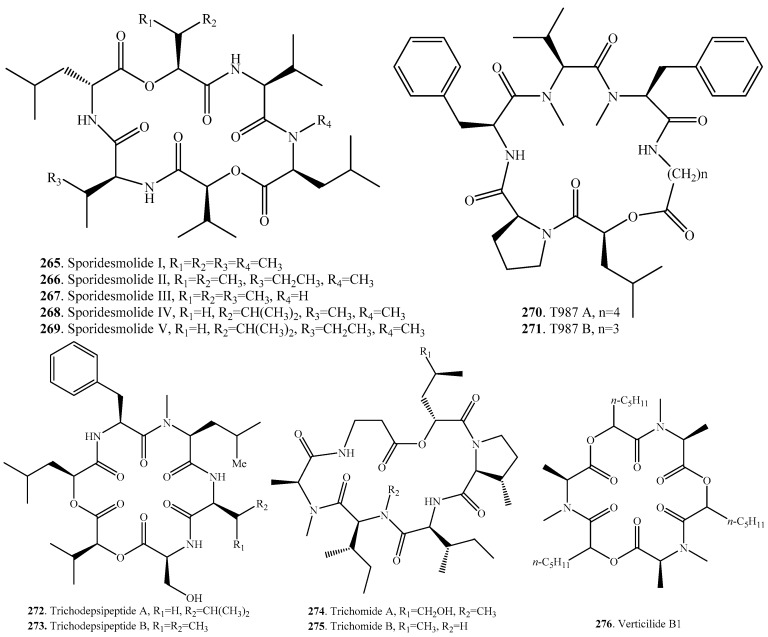
Structures of the cyclic hexadepsipeptides isolated from fungi.

Cyclic hexadepsipeptides are mainly distributed in the genera *Acremonium*, *Aspergillus*, *Beauveria*, *Cordyceps*, *Fusarium*, *Isaria*, *Nigrospora*, *Peacilomyces*, and *Verticillium*. They represent the largest class of CDPs found in fungi. Most of cyclic hexadepsipeptides belong to mycotoxins. Their occurrences and biological activities are shown in Table 4, and their structures are provided in Figure 4. The main groups of cyclic hexadepsipeptides include beauvenniatins, beauvericins, destruxins, enniatins, isaridins and isariins which have been well reviewed, respectively [16,17,18,19].

Six aspergillicins analogs **94**–**99** were isolated from *Aspergillus* sp. [83,84]. Among them, aspergillicin F (**99**) showed innate immune-modulating activity [84].

Beauvenniatins A–E (**100**–**104**), and beauvericin J (**125**) from *Acremonium* sp. BCC 28424 showed antimalaria on *Plasmodium falciparum* K1, antituberculosis on *Mycobacterium tuberculosis* H37Ra, and cytotoxic activities on cancer cell lines (KB, MCF-7, and NCI-H187) and *Vero* cells. Beauvenniatins C (**102**), D (**103**), E (**104**), and beauvericin J (**125**), containing an *N*-Me-l-Tyr residue, showed weaker activity [85].

Beauvenniatin F (**105**) was isolated from an entomogenous fungus *Fusarium proliferatum* from the cadaver of an unidentified insect collected in Tibet, and exhibited strong cytotoxicity against K562/A (adriamycin-resistant K562) cells with IC_50_ value of 3.78 μM, and autophagy-inducing activity at the concentration of 20 μM in GFP-LC3 stable HeLa cells [86]. Beauvenniatins F (**105**), G_1_ (**106**), G_2_ (**107**), G_3_ (**108**), H_1_ (**109**), H_2_ (**110**), and H_3_ (**111**) from the fungus *Acremonium* sp. BCC 2629 exhibited antibacterial activity against *Mycobacterium tuberculosis* H37Ra with MIC values in the range of 1.07–4.45 μM, and proliferation inhibitions against the human malaria parasite (*Plasmodium falciparum* K1) with IC_50_ values in the range of 3.6–3.9 μM. They also displayed cytotoxic activity toward cancer cell-lines (KB, BC, NCI-H187 cell-lines) with IC_50_ values ranging from 1.00 to 2.29 μM, as well as *Vero* cells with IC_50_ values in the range of 1.9–5.5 μM [87].

Beauvericins and allobeauvericins are a class of cyclohexadepsipeptides with core structures made of three *N*-methyl-l-phenylalanine units connected alternately with three 2-hydroxy-d-isovaleric acid residues. They were first isolated from the culture of the insect-pathogenic fungus *Beauverina bassiana* [88]. They consisted of alternating 2-hydroxy-3-methylbutanoic acid and amino acid units. The three amino acid residues are aromatic *N*-methyl-l-phenylalanines. Beauvericin (BEA, **112**) was found in in many entomophathogenic fungi such as *Beauveria bassiana*, *Isaria tenuipes* (formerly *Paecilomyces tenuipes*), *Isaria fumosorosea* (formerly *Paecilomyces fumosoroseus*), *Cordyceps cicadae*, all of these species are members of family Cordycipitaceae. BEA (**112**) has also been isolated from many *Fusarium* species (i.e, *F. acuminatum*, *F. acutatum*, *F. anthophilum*, *F. avenaceum*, *F. beoniforme*, *F. circinatum*, *F. concentricum*, *F. dlamini*, *F. equiseti*, *F. fujikuoi*, *F. globosum*, *F. guttiforme*, *F. konzum*, *F. langsethiae*, *F. longipes*, *F. nygamai*, *F. oxysprum*, *F. poae*, *F. proliferatum*, *F. pseudoanthophilum*, *F. sambucinum*, *F. semitectum*, *F. sporotrichioides*, *F. subglutinans*, *F. tricinctum*, and *F. verticilloides*). BEA was suggested as a chemotaxonomic marker of the fungi in genus *Fusarium* [17] and family Cordycipitaceae [89].

Destruxins are mainly isolated from the entomopathogenic fungus *Metarhizium anisopliae*. More than 35 destruxin analogs have been identified in this fungus [19]. Destruxin A (**141**) can induce and bind heat shock proteins (HSPs) in *Bombyx mori* Bm12 cells [90]. Most of destruxins exhibit insecticidal and phytotoxic activities. Other biological activities include antimicrobial, antitrypanosomal, cytotoxic, immunosuppressant, antiproliferative and antiviral acitivites. Destruxins act as V-ATPase inhibitors and provide a basis for the development of new drugs to against osteoporosis, cancer, or as the biological control agents [16,19]. Destrusins cause an initial tetanic paralysis, which is attributed to muscle depolarization by direct opening of Ca^2+^ channels in the membrane [16]. They can act as V-ATPase inhibitors, and modulate the antiapoptotic funcition of Bcl-xL through their inherent ability to inhibit the V-ATPase activity as a result of a caspase-independent pathway [19].

Enniatins have been isolated largely from *Fusarium* species, although they were isolated from other fungal genera, such as *Verticillium* and *Halosarpheia* [18]. About 30 enniatins have been isolated and characterized, either as a single compound or mixtures of inseparable homologs. Structurally, these depsipeptides are biosynthesized by a multifunctional enzyme, termed enniatin synthetase, and composed of six residues that alternate between *N*-methyl amino acids and hydroxylated carboxylic acids [18].

Enniatins A (**177**), A_1_ (**178**), B (**180**), B_1_ (**181**), D (**184**), E_1_ (**186**), E_2_ (**187**) and F (**188**) were isolated from the culture broth of *Fusarium* sp. FO-1305 [91]. In an enzyme assay using rat liver microsomes, they were found to inhibit acyl-CoA:cholesterol acyltransferase (ACAT) activity with IC_50_ values of 22 to 110 μM [92]. Enniatins A1 (**178**) and B1 (**181**) were found to induce apoptotic cell death and disrupt extracellular-regulated protein kinase, a mitogen-activated protein kinase associated with cell proliferation. They incorporate easily into the cell membrane as a passive channel and form action selective pores. By forming complexes with cations like K^+^, Na^+^ and Ca^2+^, enniatins evoke changes in intracellular ion concentration, disrupting cell function [18].

Enniatins H (**190**), I (**191**), and MK1688 (**199**), and beauvericin (**112**) were purified from *Fusarium oxysporum* KFCC 11363. Enniatins I (**191**) and MK1688 (**199**) inhibited the growth of cancer cell lines most strongly and had similar cytotoxic effects on the tested human cancer cell cultures [93].

Hirsutellide A (**218**), isolated from the entomopathogenic fungus *Hirsutella kobayasii*, showed antimycobacterial activity (IC_50_, 6–12 μg/mL) and antimalarial activity (IC_50_, 2.8 μg/mL) on *Plasmodium falciparum* [94].

Isarfelins A (**225**/**226**) and B (**228**) were isolated from the mycelia of *Isaria felina*. They were later identified as isarridins C1 (**225**)/C2 (**226**) and E (**228**), respectively, and exhibited antifungal activity on *Rhizoctonia solani* and *Sclerotinia sclerotiorum*, and insecticidal activity on *Leucania separata* [95].

Isoisariin B (**240**) was isolated from the entomopathogenic fungus *Beauveria felina*. This compound was active against the pest-insect *Sitophilus* spp. with an LD_50_ value of 10 μg/mL [96]. Other isariin analogs including isariins A (**231**), B (**232**), C (**233**), C2 (**234**), D (**235**), E (**236**), F2 (**237**), G1 (**238**), G2 (**239**), and isoisariin D (**241**) were identified in the fungus *Beauveria felina* [96,97,98,99].

Nodupeptide (**242**) was isolated from the gut of the insect *Riptortus pedestris*. This compound displayed insecticidal activity against rice brown planthopper (*Nilaparvata lugens*) with an LD_50_ value of 70 ng/larva, and inhibitory activity towards the drug-resistant human pathogenic bacterium *Pseudomonas aeruginosa* with the MIC value (5.0 μM) comparable to that (3.2 μM) of the positive control ciprofloxacin [100].

Paecilodepsipeptide A (also namely gliotide, **248**) was first obtained from the marine-derived fungus *Gliocladium* sp. from the alga *Durvillaea antarctica* [101], and later isolated from the insect pathogenic fungus *Paecilomyces cinnamomeus* BCC 9616 [102]. This compound exhibited antimalarial activity on *Plasmodium falciparum* K1 and cytotoxic activity on KB and BC cell lines [102].

Pseudodestruxins A (**249**) and B (**250**) were obtained from the coprophilous fungus *Nigrosabulum globosum* isolated from sheep dung. Both had antibacterial activity on *Bacillus subtilis* and *Staphylococcus aureus* [103].

Roseotoxin B (**259**) from *Trichothecium roseum* improved allergic contact dermatitis through a unique anti-inflammatory mechanism involving excessive activation of autophagy in activated T lymphocytes [104].

Trichodepsipeptides A (**272**) and B (**273**), and guangomide A (**214**) were isolated from the filamentous fungus *Trichothecium* sp. (MSX 51320) [105]. Guangomide A (**214**) showed weak antibacterial activity on *Staphylococcus epidermids* and *Enterococcus durans* [106].

Trichomides A (**274**) and B (**275**) were isolated from *Trichothecium roseum*. Trichomide A (**274**) decreased the expression of Bcl-2 and increased that of Bax, with mild or negligible effects on the levels of p-Akt, CD25, and CD69. It provided valuable information for lead structure optimization of the novel immunosuppressant [107].

## 6. Cyclic Heptadepsipeptides

**Figure 5:**
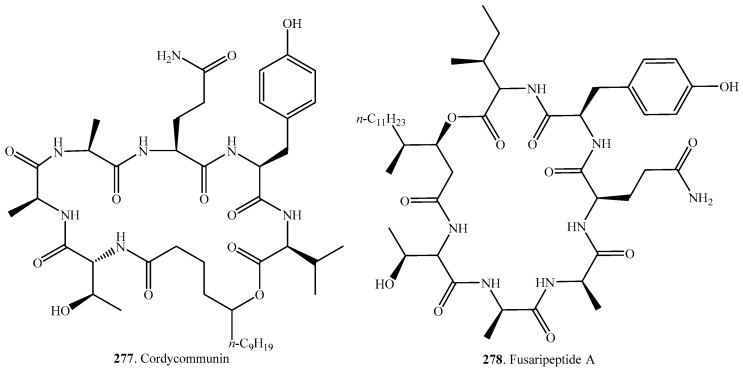

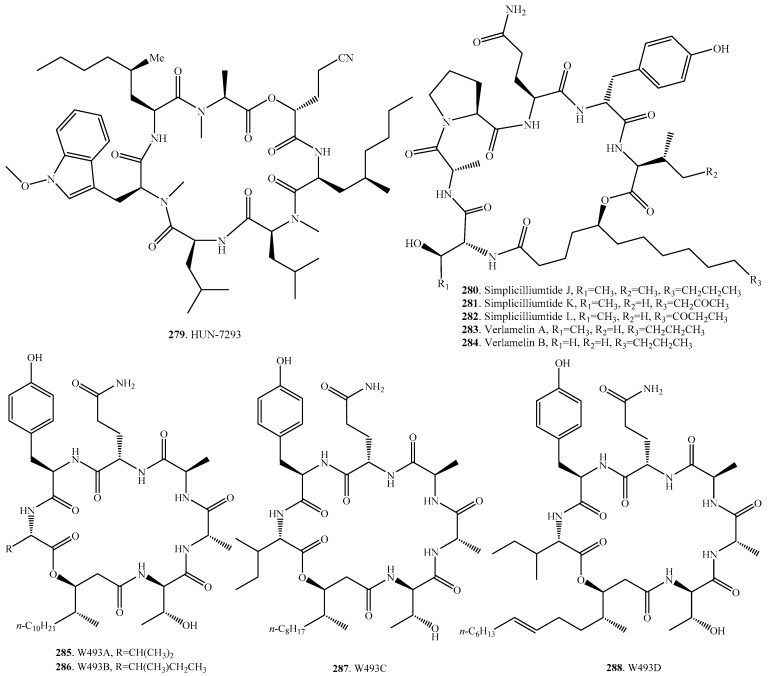
Structures of the cyclic heptadepsipeptides isolated from fungi.

The occurrences and biological activities of fungal cyclic heptadepsipeptides are shown in Table 5, and their structures are provided in Figure 5.

Cordycommunin (**277**) was obtained from the insect pathogenic fungus *Ophiocordyceps communis* BCC16475. This compound exhibited inhibitory activity on *Mycobacterium tuberculosis* H37Ra. It also showed weak cytotoxic activity on KB cells [180].

Fusaripeptide A (**278**) was obtained from the endophytic fungus *Fasarium* sp. from the roots of *Mentha longifolia* L. growing in Saudi Arabia. It exhibited antifungal, anti-malarial and cytotoxic activities [181].

Simplicilliumtides J (**280**), K (**281**), L (**282**) and verlamelins A (**283**) and B (**284**) were isolated from the deep-sea-derived fungus *Simplicillium obclavatum* EIODSF 020. Simplicilliumtides J (**280**), and verlamelins A (**283**) and B (**284**) showed antifungal activity toward *Aspergillus versicolor* and *Curvularia australiensis*, and also had obvious antiviral activity on HSV-1 with IC_50_ values of 14.0, 16.7, and 15.6 μM, respectively [182]. Verlamelins A (**283**) and B (**284**) were obtained from the entomopathogenic fungus *Lecanicillium* sp. (formerly *Verticillium lecanii*) isolated from a chillie trips cadaver. They showed antifungal activity against plant pathogenic fungi [183].

W493 A (**285**), B (**286**), C (**287**) and D (**288**) were obtained from the endophytic fungus *Fusarium* sp. isolated from the mangrove plant *Ceriops tagal*. Both W493 A (**285**) and B (**286**) exhibited moderate activity against the fungus *Cladosporium cladosporiodes* and weak antitumor activity against the human ovarian cancer cell line A2780 [184]. W493 A and B were also isolated from *Fusarium* sp. and showed strong antifungal activity against *Venturia inaequalis*, *Monilinia mali*, and *Cochliobolus miyabeanus* [185].

## 7. Cyclic Octadepsipeptides

**Figure 6:**
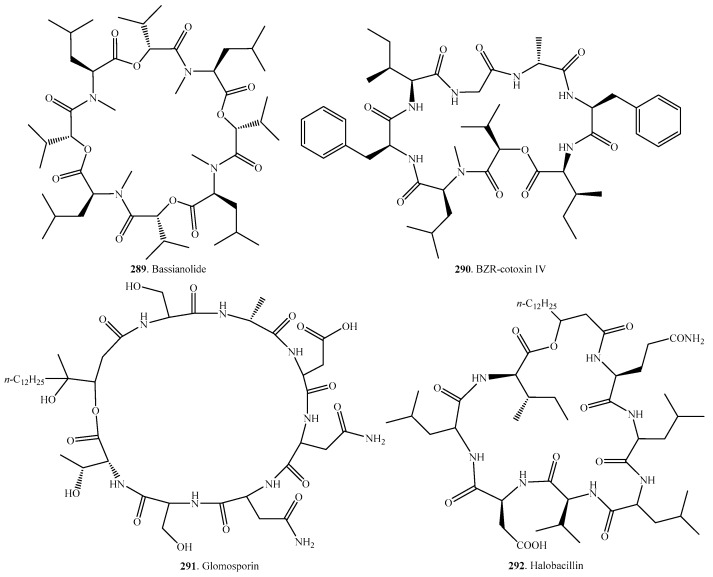

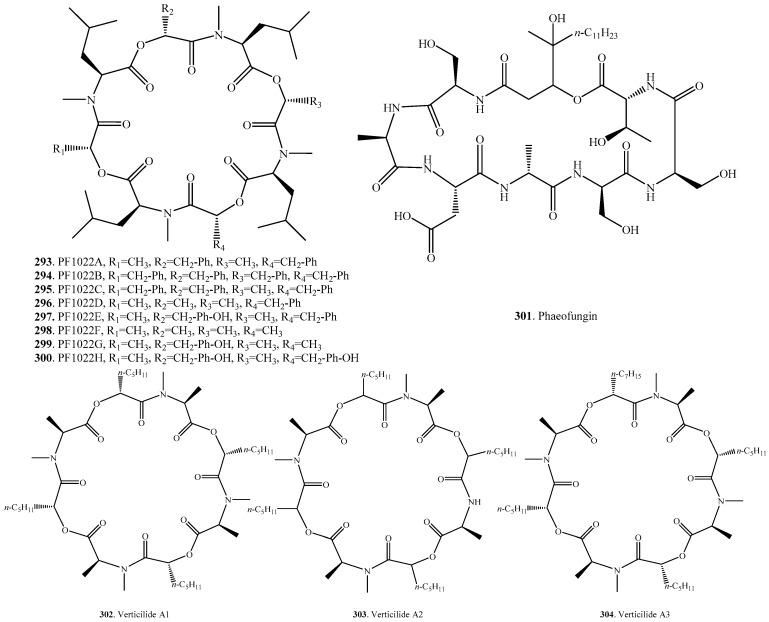
Structures of the cyclic octadepsipeptides isolated from fungi.

The occurrences and biological activites of reported fungal cyclic octadepsipeptides are listed in Table 6, and their structures are shown in Figure 6.

Bassianolide (**289**) was isolated from *Beauveria bassiana*, *Lecanicilium* sp. (formerly *Verticillium lecanii*), and *Xylaria* sp. BCC1067 to display insecticidal, cytotoxic and anthelmintic acitivities [187,188,189]. Synthesis of bassianolide (**289**) was succeeded, and this compound showed antitumor activity by inducing G0/G1 arrest in MDA-MB 231 breast cancer cells [190].

The broad-spectrum anthelimintic cyclic octadepsipeptides PF1022A (**293**), PF1022B (**294**), PF1022C (**295**), PF1022D (**296**), PF1022E (**297**), PF1022F (**298**), PF1022G (**299**) and PF1022H (**300**) were isolated from the endophytic fungus *Rosellinia* sp. PF1022 from the leaves of *Camellia japonica* [191,192]. The action mode of PF1022A (**293**) appeared to be complex, having at least two different targets, a latrophilin-like receptor, and a Ca^2+^-activated K^+^ channel [193]. The synthesis and biosynthesis of PF1022A (**293**) have also been studied in detail [194,195]. These metabolites were used as starting points to generate semisynthetic derivatives among which emodepside has been developed as the commercial anthelmintic agent Emodepside against gastrointestinal and extraintestinal parasites [193].

Phaeofungin (**301**), which was isolated from the endophytic fungus *Phaeosphaeria* sp. from living stems and leaves of *Sedum* sp. (Crassulaceae), was discovered by application of reverse genetics technology, using the *Candida albicans* fitness test (*Ca*FT). This compound caused ATP release in wild-type *Candida albicans* strains. It showed modest antifungal activity with the MICs for *Candida albicans*, *Aspergillus fumigatus*, and *Trichophyton mentagrophytes* as 16, 8 and 4 µg/mL, respectively [196].

Verticilides A_1_ (**302**), A_2_ (**303**) and A_3_ (**304**) were isolated from *Verticillium* sp. FKI-2679. These compounds showed inhibitory activity on acyl-CoA:cholesterol acyltransferase (ACAT) in a cell-based assay using ACAT1- and ACAT2-expressing CHO cells [179].

## 8. Cyclic Nonadepsipeptides

**Figure 7:**
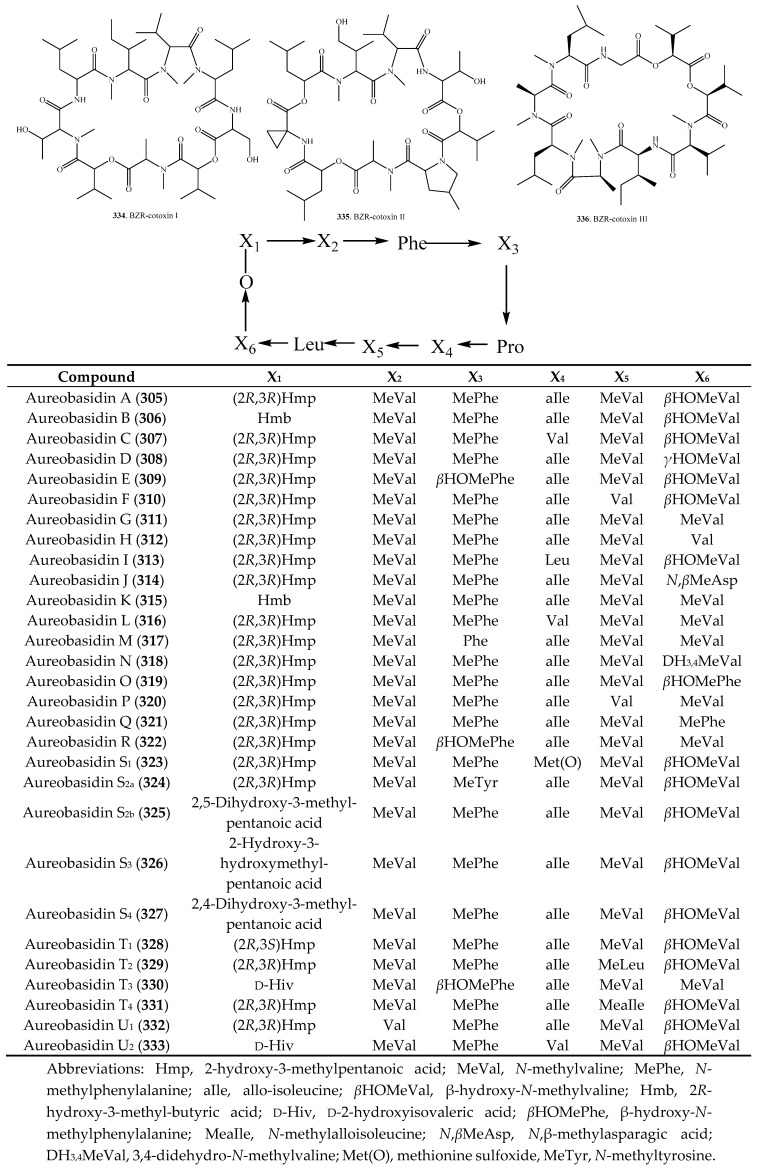

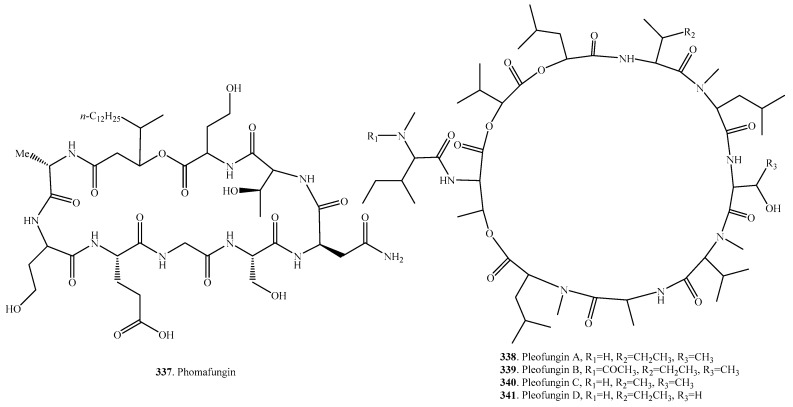
Structures of the cyclic nonadepsipeptides isolated from fungi.

The origins and biological activities of fungal cyclic nonadepsipeptides are listed in Table 7, and their structures are provided in Figure 7. Aureobasins were isolated from the black yeast *Aureobasidium pullulans* R106 from the leaf collected at Tsushima of Japan. They are composed of one hydroxylated carboxylic acid and eight amino acids, and 29 aureobasidin analogs (**305**–**333**) have been isolated from this fungus [203,204,205,206]. They showed good in vitro activity against all *Candida* species and *Cryptococcus neoformans*, in vivo activity against murine systemic candidiasis, and had low toxicity. They also showed inhibitory activity on inositol phosphorylceramide synthase [207].

BZR-cotoxin I (**334**) was isolated from plant pathogenic fungus *Bipolaris zeicola* [208] and endophytic fungus *Bipolaris sorokiniana* LK12 [198]. It had moderate anti-lipid peroxidation and urease activities [198]. Pleofungins A (**338**), B (**339**), C (**340**) and D (**341**) were identified from *Phoma* sp. SANK 13899 from a soil sample collected at Tokyo of Japan. It is a rare case where a CDP contains three subsequent lactone bonds. These CDPs showed inhibitory activity on inositol phosphorylcermide synthase [209,210].

## 9. Cyclic Decadepsipeptides

**Figure 8:**
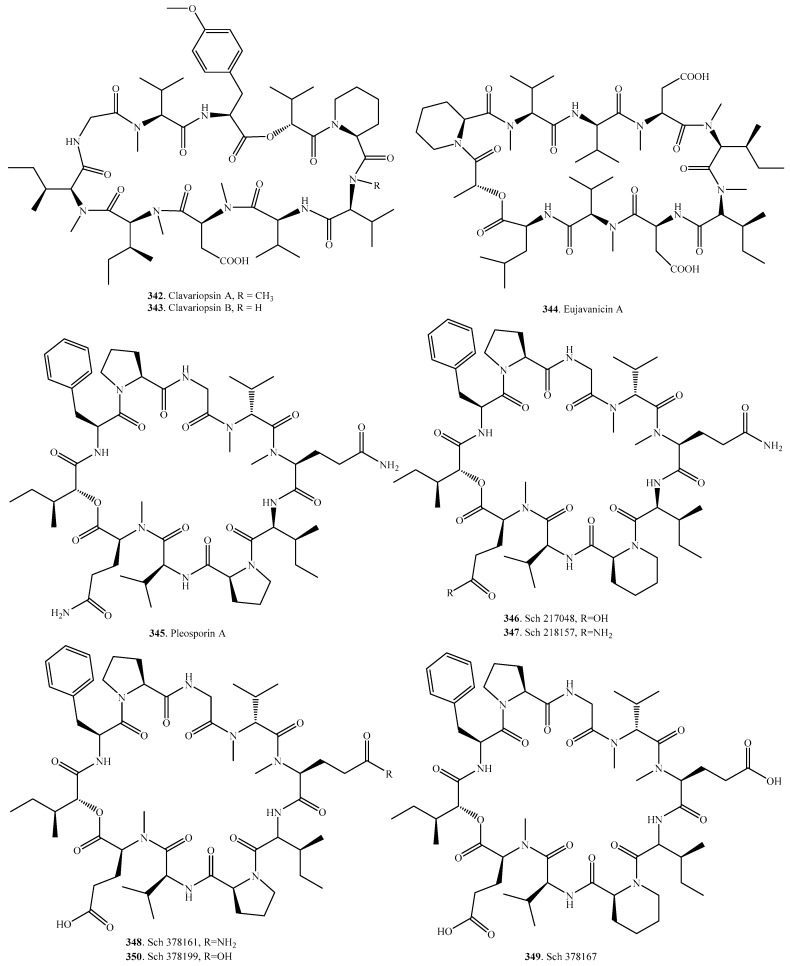
Structures of the cyclic decadepsipeptides isolated from fungi.

The occurrences and biological activities of fungal cyclic decadepsipeptides are shown in Table 8, and their structures are provided in Figure 8. Only eight cyclic decadepsipeptides have been identified in fungi. Clavariopsins A (**342**) and B (**343**) were produced by an aquatic hyphomycetes, *Clavariopsis aquatic* [217]. Both showed antifungal activity by inhibiting fungal cell wall biosynthesis [218]. Four tachykinin (NK_2_) receptor inhibitors, SCH 217048 (**346**), SCH 378161 (**348**), SCH 378167 (**349**) and SCH 378199 (**350**) were isolated from a taxonomically unidentified fungus. They were selective and competitive receptor antagonists of the human NK_2_ receptor [219]. Both Sch 217048 (**346**) and Sch 378161 (**348**) were also isolated from the freshwater fungus *Clohesyomyces aquaticus* [220].

## 10. Cyclic Tridecadepsipeptides

**Figure 9:**
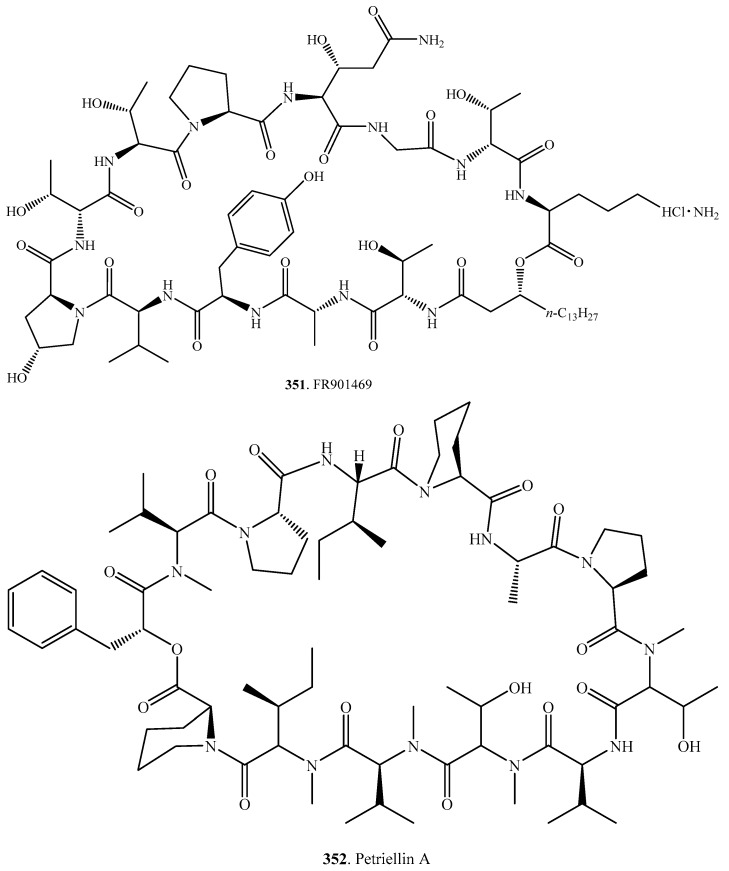
Structures of the cyclic tridecadepsipeptides isolated from fungi.

Up to now, only two tridecadepsipepitdes namely FR901469 (**351**) and petriellin A (**352**) have been identified in fungi [224]. Their structures are shown in Figure 9. FR901469 (**351**) was isolated from an unidentified fungus No.11243. This compound displayed antifungal activity by inhibiting 1,3-β-glucan synthase with an IC_50_ value of 0.05 μg/mL [224]. Petriellin A (**352**) was obtained from the coprophilous fungus *Petriella sordida*. It exhibited antifungal activity against *Ascobolus furfuraceus* (NRRL 6460) and *Sordaria fimicola* (NRRL 6459) [225].

## 11. Conclusions and Future Perspectives

In this review, we describe the chemistry and biology of the CDPs discovered from fungi during the past 50 years. It is worth mentioning that more and more CDPs have been isolated from plant endophytic and marine-derived fungi which indicate that plant-derived endophytic and marine-drived fungi are the mines of biologically active natural products [10,13,226,227,228]. Some invertebrate derived CDPs (e.g., from sponge origin) are actually synthesized by the symbiotic microorganisms [229]. In addition, some minor or new CDPs have been identified in fungi with the application of new techniques such as LC-MS/MS [230], reverse genetics [196], genomics [138], epigenetic manipulation [62], and combinatorial biosynthesis [231,232].

Fungal CDPs are mainly reported from the genera *Acremonium*, *Alternaria*, *Aspergillus*, *Beauveria*, *Fusarium*, *Isaria*, *Metarhizium*, *Penicillium*, and *Rosellina*. Among the CDPs, cyclic hexadepsipeptides account for the largest proportion. Most of them are mycotoxins such as beauvenniatins, beauvericins, destruxins, and enniatins [16,17,18,19]. Compared to the cyclic peptides only with amide bonds [14,15], the ring size of CDPs seems to be smaller.

Many fungal CDPs such as aureobasidins (**305**–**333**), beauvericin (**112**), paecilodepsipeptide A (**248**) and sansalvamide A (**87**), show an interesting spectrum of biological activities, can be used as either drug candidates or lead compounds for drug development [21]. Their potential applications as antitumor agents, herbicides, antimicrobials, and insecticidals have attracted considerable interest within the pharmaceutical and agrochemical companies [19,233,234,235]. Chemical syntheses have been achieved for many bioactive CDPs such as aspergillicin F (**99**) [84], enniatin B (**180**) [236], PF1022A (**293**) [194], and zygosporamide (**88**) [237]. The biosynthetic pathways of some fungal CDPs such as beauvericin (**112**) [238], enniatin (**177**) [239], fusaristatin A (**51**) and W493 B (**286**) [240], verlamelin (**283**) [241] have also been revealed. They were considered to be biosynthesized by the non-ribosomal peptide synthetases (NRPS) [231].

Some fungal CDPs are currently in clinical use or have entered human clinical trials as antibiotic or anticancer agents. Some have been developed into commercial products [18,19,22]. The noteworthy example is the anthelmintic agent emodepside which is a semisynthetic derivative of PF1022A (**293**), a cyclic octadepsipeptide from the endophytic fungus *Rosellina* sp. PF1022 derived from the leaves of *Camellia japonica* [191]. Emodepside binds to a presynaptic latrophilin receptor and interacts with a calcium-activated potassium channel. Both modes of action cause paralysis and death of the nematode [242]. It is employed against gastrointestinal and extraintestinal parasites such as nematodes in veterinary medicine [193]. Another example is fusafungine, a mixture of enniatins, which is an antibacterial for the treatment of rhinosinusitis in nasal spray [18]. However, fusafungine has been recently withdrawn from the EU market since enniatins have been previously identified as mycotoxins which pose a potential health hazard on humans or animals [243,244,245]. The third example is the direct application of destruxins as insecticidal agents [19]. Destruxins were isolated from a variety of fungi such as *Metarrhizium anisopliae* [16], *Beauveria felina* [123], and *Ophiocordyceps coccidiicola* [128]. With the increasing understanding of the biosynthetic pathways of some fungal CDPs, we can rationally design bioengineering approaches such as chemoenzymatics, mutasynthesis, site-directed mutagenesis, and combinatorial biosynthesis. We may be able to effectively not only increase the yields of bioactive CDPs, but also block the biosynthesis of some toxic depsipeptides [231,246].

## Figures and Tables

**Table 1:** Fungal cyclic tridepsipeptides and their biological activities.

Name	Fungus and Its Origin	Biological Activity	References
Acremolide A (**1**)	Marine-derived fungus *Acremonium* sp. MST-MF588a from an estuarine sediment sample	-	[23]
Acremolide B (**2**)	Marine-derived fungus *Acremonium* sp. MST-MF588a from an estuarine sediment sample	-	[23]
Acremolide C (**3**)	Marine-derived fungus *Acremonium* sp. MST-MF588a from an estuarine sediment sample	-	[23]
Acremolide D (**4**)	Marine-derived fungus *Acremonium* sp. MST-MF588a from an estuarine sediment sample	-	[23]
Calcaripeptide A (**5**)	Marine-derived fungus *Calcarisporium* sp. KF525 from a water sample collected in the German Wadden Sea	-	[24]
Calcaripeptide B (**6**)	Marine-derived fungus *Calcarisporium* sp. KF525 from a water sample collected in the German Wadden Sea	-	[24]
Calcaripeptide C (**7**)	Marine-derived fungus *Calcarisporium* sp. KF525 from a water sample collected in the German Wadden Sea	-	[24]
HA 23 (**8**)	*Fusarium* sp. CANU-HA23	-	[25]
PM181110 (**9**)	Endophytic fungus *Phomopsis glabrae* from the leaves of *Pongamia pinnata*	Cytotoxic activity	[26]
Stereocalpin A (**10**)	Endophytic fngus *Ramalina terebrata* from the Antarctic lichen *Stereocaulon alpinum*	Cytotoxic activity	[27]

**Table 2:** Fungal cyclic tetradepsipeptides and their biological activities.

Name	Fungus and Its Origin	Biological Activity	References
15G256γ (**11**)	*Hypoxylon oceanicum* LL-15G256	Antifungal activity	[29,30]
15G256δ (**12**)	*Hypoxylon oceanicum* LL-15G256	Antifungal activity	[29,30]
15G256ε (**13**)	*Hypoxylon oceanicum* LL-15G256	Antifungal activity	[29,30]
AM-toxin I (**14**)	*Alternaria mali*	Phytotoxic activity	[32,34]
AM-toxin II (**15**)	*Alternaria mali*	Phytotoxic activity	[33,34]
AM-toxin III (**16**)	*Alternaria mali*	Phytotoxic activity	[32,33,34]
Angolide (**17**)	*Pithomyces* sp. IMI 101184	-	[48]
Aspergillipeptide A (**18**)	*Aspergillus* sp. SCSGAF 0076 from China South Sea gorgonian *Melitodes squamata*	-	[35]
Aspergillipeptide B (**19**)	*Aspergillus* sp. SCSGAF 0076 from China South Sea gorgonian *Melitodes squamata*	-	[35]
Aspergillipeptide C (**20**)	*Aspergillus* sp. SCSGAF 0076 from China South Sea gorgonian *Melitodes squamata*	Antifouling activity against *Bugula neritina* larvae settlement	[35]
Beauveriolide I (**21**)	*Beauveria* sp.	Insecticidal activity on *Spodoptera litura* and *Callosobruchus chinensis*	[36]
Beauveriolide II (**22**)	*Beauveria* sp.	-	[36]
Beauveriolide III (**23**)	*Beauveria* sp. FO-6979	-	[37]
	-	Selective inhibition of sterol *O*-acyltransferase 1	[39]
Beauveriolide IV (**24**)	*Beauveria* sp. FO-6979	-	[38]
Beauveriolide V (**25**)	*Beauveria* sp. FO-6979	-	[38]
Beauveriolide VI (**26**)	*Beauveria* sp. FO-6979	-	[38]
Beauveriolide VII (**27**)	*Beauveria* sp. FO-6979	-	[38]
Beauveriolide VIII (**28**)	*Beauveria* sp. FO-6979	-	[38]
Beauverolide A (**29**)	Entomopathogenic fungus *Beauveria bassiana* from a pupa of the Gum Emperor moth *Antheraea eucalypti*	Insecticidal activity	[49]
Beauverolide B (**30**)	Entomopathogenic fungus *Beauveria bassiana* from a pupa of the Gum Emperor moth *Antheraea eucalypti*	Insecticidal activity	[49]
Beauverolide Ba = Beauverilide A (**31**)	*Beauveria bassiana*	-	[50]
	Entomopathogenic fungus *Beauveria bassiana* from a pupa of the Gum Emperor moth *Antheraea eucalypti*	Anti-aging activity; Insecticidal activity	[51,52]
Beauverolide C (**32**)	Entomopathogenic fungus *Beauveria bassiana* from a pupa of the Gum Emperor moth *Antheraea eucalypti*	Insecticidal activity	[49]
Beauverolide Ca (**33**)	*Beauveria bassiana*	-	[50]
Beauverolide D (**34**)	Entomopathogenic fungus *Beauveria bassiana* from a pupa of the Gum Emperor moth *Antheraea eucalypti*	Insecticidal activity	[49]
Beauverolide E (**35**)	Entomopathogenic fungus *Beauveria bassiana* from a pupa of the Gum Emperor moth *Antheraea eucalypti*	Insecticidal activity	[49]
Beauverolide Ea (**36**)	*Beauveria bassiana*	-	[49]
Beauverolide F (**37**)	Entomopathogenic fungus *Beauveria bassiana* from a pupa of the Gum Emperor moth *Antheraea eucalypti*	Insecticidal activity	[49]
Beauverolide Fa = Beauveriolide IX (**38**)	*Beauveria bassiana*	-	[49]
	*Beauveria* sp. FO-6979	-	[38]
Beauverolide H (**39**)	*Beauveria bassiana*	-	[53]
Beauverolide I (**40**)	*Beauveria bassiana*	-	[53]
Beauverolide Ja (**41**)	*Beauveria bassiana*	-	[50]
Beauverolide Ka (**42**)	*Beauveria bassiana*	-	[50]
Beauverolide L (**43**)	*Beauveria tenella* and *Paecilomyces fumosoroseus*	-	[54]
Beauverolide La (**44**)	*Beauveria tenella* and *Paecilomyces fumosoroseus*	-	[54]
Beauverolide M (**45**)	*Beauveria bassiana*	-	[55]
Beauverolide N (**46**)	*Beauveria bassiana*	-	[55]
Beauverolide P (**47**)	*Beauveria bassiana*	-	[55]
Chaetomiamide A (**48**)	Endophytic fungus *Chaetomium* sp. from the roots of *Cymbidium goeringii*	-	[56]
Clavatustide A (**49**)	*Aspergillus clavatus*	Cytotoxic activity	[40]
Clavatustide B (**50**)	*Aspergillus clavatus*	Cytotoxic activity	[40,41]
Fusaristatin A (**51**)	Endophytic fungus *Fusarium* sp. YG-45	Cytotoxic activity	[42]
	Endophytic fungus *Fusarium decemcellulare* LG53	Antifungal activity	[43]
Fusaristatin B (**52**)	Endophytic fungus *Fusarium* sp. YG-45	Weak activity against topoisomerases I and II; Cytotoxic activity	[42]
Stevastelin A (**53**)	*Penicillium* sp. NK374186 from the soil collected in Niigata of Japan	Immunosuppressant by inhibiting dual-specificity protein phosphatase	[44,45,46]
Stevastelin A_3_ (**54**)	*Penicillium* sp. NK374186 from the soil collected in Niigata of Japan	Immunosuppressant by inhibiting dual-specificity protein phosphatase	[46]
Stevastelin B (**55**)	*Penicillium* sp. NK374186 from the soil collected in Niigata of Japan	Immunosuppressant by inhibiting dual-specificity protein phosphatase	[44,45,57]
Stevastelin B3 (**56**)	*Penicillium* sp. NK374186 from the soil collected in Niigata of Japan	Immunosuppressant by inhibiting dual-specificity protein phosphatase	[44,45]
Stevastelin C3 (**57**)	*Penicillium* sp. NK374186 from the soil collected in Niigata of Japan	Immunosuppressant by inhibiting dual-specificity protein phosphatase	[44]
Stevastelin D3 (**58**)	*Penicillium* sp. NK374186 from the soil collected in Niigata of Japan	Immunosuppressant by inhibiting dual-specificity protein phosphatase	[46]
Stevastelin E3 (**59**)	*Penicillium* sp. NK374186 from the soil collected in Niigata of Japan	Immunosuppressant by inhibiting dual-specificity protein phosphatase	[46]

**Table 3:** Fungal cyclic pentadepsipeptides and their biological activities.

Name	Fungus and Its Origin	Biological Activity	References
Alternaramide (**60**)	Marine-derived *Alternaria* sp. SF-5016	Weak antibiotic activity	[58]
	-	Anti-inflammatory activity	[59]
Aselacin A (**61**)	*Acremonium* sp.	Inhibitory activity on binding of endothelin to its receptor	[60,61]
Aselacin B (**62**)	*Acremonium* sp.	Inhibitory activity on binding of endothelin to its receptor	[60,61]
Aselacin C (**63**)	*Acremonium* sp.	Inhibitory activity on binding of endothelin to its receptor	[60,61]
Brevigellin (**64**)	*Penicillium brevicompactum*	-	[76]
Colisporifungin (**65**)	*Colispora cavincola*	Antifungal activity	[77]
EGM-556 (**66**)	*Microascus* sp.	Histone deacetylase inhibitor	[62]
Hikiamide A (**67**)	*Fusarium* sp. TAMA 456 from a rotten wood sample	Induction of adipocyte differentiation and mRNA expression	[63]
Hikiamide B (**68**)	*Fusarium* sp. TAMA 456 from a rotten wood sample	Induction of adipocyte differentiation and mRNA expression	[63]
Hikiamide C (**69**)	*Fusarium* sp. TAMA 456 from a rotten wood sample	Induction of adipocyte differentiation and mRNA expression	[63]
JBIR-113 (**70**)	Sponge-derived *Penicillium* sp. fS36	-	[64]
	Endophytic fungus *Penicillium brasilianum*	Weak antiparasitic activity	[65]
JBIR-114 (**71**)	Sponge-derived *Penicillium* sp. fS36	-	[64]
JBIR-115 (**72**)	Sponge-derived *Penicillium* sp. fS36	-	[64]
Leualacin (**73**)	*Hapsidospora irregularis*	Calcium channel blocker	[66,67]
Leualacin B (**74**)	*Hapsidospora irregularis*	-	[68]
Leualacin C (**75**)	*Hapsidospora irregularis*	-	[68]
Leualacin D (**76**)	*Hapsidospora irregularis*	-	[68]
Leualacin E (**77**)	*Hapsidospora irregularis*	-	[68]
Leualacin F (**78**)	*Hapsidospora irregularis*	Elicitation of calcium influx	[68]
Leualacin G (**79**)	*Hapsidospora irregularis*	-	[68]
MBJ-0110 (**80**)	*Penicillium* sp. f25267	-	[78]
Neo-*N*-methylsansalvamide A (**81**)	*Fusarium solani* KCCM90040	Cytotoxic activity	[79]
*N*-methylsansalvamide (**82**)	Marine-derived fungus *Fusarium* sp. CNL-619.	Cytotoxic activity	[80]
Petrosifungin A (**83**)	Marine-derived *Penicillium brevicompactum*	-	[81]
Petrosifungin B (**84**)	Marine-derived *Penicillium brevicompactum*	-	[81]
Phomalide (**85**)	*Phoma lingam*	Phytotoxic activity	[70]
Pithomycolide (**86**)	*Pithomyces chatatum*	-	[82]
Sansalvamide A (**87**)	Marine-derived fungus *Fusarium* sp.	Cytotoxic, topoisomerase I inhibitory, and antitumor activities	[71,72]
Zygosporamide (**88**)	Marine-derived fungus *Zygosporium masonii*	Cytotoxic activity against SF-268 and RXF 393 cell lines	[75]

**Table 4:** Fungal cyclic hexadepsipeptides and their biological activities.

Name	Fungus and Its Origin	Biological Activity	References
1962A (**89**)	Unidentified fungus from *Kandelia candel* leaf	Weak activity against human breast cancer MCF-7 cells	[108]
1962B (**90**)	Unidentified fungus from *Kandelia candel* leaf	-	[108]
Allobeauvericin A (**91**)	*Peacilomyces tenuipes* BCC 1614	-	[109]
Allobeauvericin B (**92**)	*Peacilomyces tenuipes* BCC 1614	-	[109]
Allobeauvericin C (**93**)	*Peacilomyces tenuipes* BCC 1614	-	[109]
Aspergillicin A (**94**)	*Aspergillus carneus* from an estuarine sediment	-	[83]
Aspergillicin B (**95**)	*Aspergillus carneus* from an estuarine sediment	-	[83]
Aspergillicin C (**96**)	*Aspergillus carneus* from an estuarine sediment	-	[83]
Aspergillicin D (**97**)	*Aspergillus carneus* from an estuarine sediment	-	[83]
Aspergillicin E (**98**)	*Aspergillus carneus* from an estuarine sediment	-	[83]
Aspergillicin F (**99**)	*Aspergillus* sp.	Innate immune-modulating activity	[84]
Beauvenniatin A (**100**)	*Acremonium* sp. BCC 28424	Antimalaria, antituberculosis and cytotoxic activities	[85]
Beauvenniatin B (**101**)	*Acremonium* sp. BCC 28424	Antimalaria, antituberculosis and cytotoxic activities	[85]
	Entomogenous fungus *Fusarium proliferatum* from the cadaver of an unidentified insect collected in Tibet	-	[86]
Beauvenniatin C (**102**)	*Acremonium* sp. BCC 28424	Antimalaria, antituberculosis and cytotoxic activities	[85]
Beauvenniatin D (**103**)	*Acremonium* sp. BCC 28424	-	[85]
Beauvenniatin E (**104**)	*Acremonium* sp. BCC 28424	Antimalaria, antituberculosis and cytotoxic activities	[85]
Beauvenniatin F (**105**)	*Acremonium* sp. BCC 2629	Antituberculosis, anti-human malaria, and cytotoxic activities	[87]
	Entomogenous fungus *Fusarium proliferatum*	Cytotoxic and autophagy-inducing activities	[86]
Beauvenniatin G_1_ (**106**)	*Acremonium* sp. BCC 2629	Antituberculosis, anti-human malaria, and cytotoxic activities	[87]
Beauvenniatin G_2_ (**107**)	*Acremonium* sp. BCC 2629	Antituberculosis, anti-human malaria, and cytotoxic activities	[87]
Beauvenniatin G_3_ (**108**)	*Acremonium* sp. BCC 2629	Antituberculosis, anti-human malaria, and cytotoxic activities	[87]
Beauvenniatin H_1_ (**109**)	*Acremonium* sp. BCC 2629	Antituberculosis, anti-human malaria, and cytotoxic activities	[87]
Beauvenniatin H_2_ (**110**)	*Acremonium* sp. BCC 2629	Antituberculosis, anti-human malaria, and cytotoxic activities	[87]
Beauvenniatin H_3_ (**111**)	*Acremonium* sp. BCC 2629	Antituberculosis, anti-human malaria, and cytotoxic activities	[87]
Beauvericin (**112**)	*Acremonium* sp. BCC 28424	Antimalaria, antituberculosis and cytotoxic activities	[85]
	*Aspergillus terreus* No. GX7-3B	*In vitro* acetylcholinesterase inhibitory activity with an IC_50_ value of 3.09 μM	[110]
	*Beauverina bassiana*	-	[88]
	*Beauveria bassiana* ATCC 7159	-	[111]
	Parasitic fungus *Cordyceps cicadae* on the larvae of *Cicada flammat*	Anti-hepatoma activity	[112]
	Endophytic fungus *Fusarium redolens* from the rhizomes of *Dioscorea zingziberensis*	Antibacterial activity	[113]
Beauvericin A (**113**)	Insect pathogenic fungus *Peacilomyces tenuipes* BCC 1614	Antimycobacterial and antiplasmodial activities	[109,114]
	Parasitic fungus *Cordyceps cicadae* on the larvae of *Cicada flammat*	Anti-hepatoma activity	[112]
Beauvericin B (**114**)	*Peacilomyces tenuipes* BCC 1614	-	[109]
Beauvericin C (**115**)	*Peacilomyces tenuipes* BCC 1614	-	[109]
Beauvericin D (**116**)	*Beauveria* sp. FKI-1366	Antifungal activity	[115]
Beauvericin E (**117**)	Parasitic fungus *Cordyceps cicadae* on the larvae of *Cicada flammat*	Anti-hepatoma activity	[112]
	*Beauveria* sp. FKI-1366	Antifungal activity	[115]
Beauvericin F (**118**)	*Beauveria* sp. FKI-1366	Antifungal activity	[115]
Beauvericin G_1_ (**119**)	*Beauveria bassiana* ATCC 7159	Cytotoxic and antihaptotactic activities	[111]
Beauvericin G_2_ (**120**)	*Beauveria bassiana* ATCC 7159	Cytotoxic and antihaptotactic activities	[111]
Beauvericin G_3_ (**121**)	*Beauveria bassiana* ATCC 7159	Cytotoxic and antihaptotactic activities	[111]
Beauvericin H_1_ (**122**)	*Beauveria bassiana* ATCC 7159	Cytotoxic and antihaptotactic activities	[111]
Beauvericin H_2_ (**123**)	*Beauveria bassiana* ATCC 7159	Cytotoxic and antiapoptotic activities	[111]
Beauvericin H_3_ (**124**)	*Beauveria bassiana* ATCC 7159	Cytotoxic and antiapoptotic activities	[111]
Beauvericin J (**125**)	*Acremonium* sp. BCC 28424	-	[85]
	Parasitic fungus *Cordyceps cicadae* on the larvae of *Cicada flammat*	Anti-hepatoma activity	[112]
Bursaphelocide A (**126**)	Unidentified fungus strain D1084	Nematicidal activity	[116]
Bursaphelocide B (**127**)	Unidentified fungus strain D1084	Nematicidal activity	[116]
Cardinalisamide A (**128**)	Insect pathogenic fungus *Cordyceps cardinalis* NBRC 103832	Antitrypanosomal activity	[117]
Cardinalisamide B (**129**)	Insect pathogenic fungus *Cordyceps cardinalis* NBRC 103832	Antitrypanosomal activity	[117]
Cardinalisamide C (**130**)	Insect pathogenic fungus *Cordyceps cardinalis* NBRC 103832	Antitrypanosomal activity	[117]
Conoideocrellide A (**131**)	Insect pathogenic fungus *Conoideocrella tenuis* BCC 18627	-	[118]
Cordycecin A (**132**)	Parasitic fungus *Cordyceps cicadae* on the larvae of *Cicada flammat*	-	[112]
Desmethyldestruxin A (**133**)	Entomopathogenic fungus *Metarhizium anisopliae*	Insecticidal activity	[119]
Desmethyldestruxin B (**134**)	Entomopathogenic fungus *Metarhizium anisopliae*	Insecticidal activity	[120]
	*Alternaria brassice*	-	[121]
Desmethyldestruxin B_2_ (**135**)	Entomopathogenic fungus *Metarhizium anisopliae*	Suppressing hepatitis B virus surface antigen production in human hepatoma cells	[122]
Desmethyldestruxin C (**136**)	Entomopathogenic fungus *Metarhizium anisopliae*	Insecticidal activity	[119]
Desmethylisaridin C_1_ (**137**)	*Beauveria felina* EN-135	Antibacterial activity on *Escherichia coli* with an MIC value of 8 μg/mL	[99]
Desmethylisaridin C_2_ (**138**)	*Beauveria felina*	Anti-inflammatory activity	[123]
Desmethylisaridin E (**139**)	*Beauveria felina*	Anti-inflammatory activity	[123]
Desmethylisaridin G (**140**)	*Beauveria felina* EN-135	-	[99]
Destruxin A (**141**)	*Alternaria linicola*	Phytotoxic activity	[124]
	*Beauveria felina*	-	[123]
	*Beauveria felina* EN-135	-	[125]
	Entomopathogenic fungus *Metarhizium anisopliae*	-	[126,127]
	Insect pathogenic fungus *Ophiocordyceps coccidiicola* NBRC 100683	Antitrypanosomal activity on *Trypanosoma brucei* with an IC_50_ value of 0.33 μg/mL	[128]
Destruxin A_1_ (**142**)	Entomopathogenic fungus *Metarhizium anisopliae*	-	[126]
Destruxin A_2_ (**143**)	Entomopathogenic fungus *Metarhizium anisopliae*	-	[126]
Destruxin A_3_ (**144**)	Entomopathogenic fungus *Metarhizium anisopliae*	Insecticidal activity	[119]
Destruxin A_4_ (**145**)	*Aschersonis* sp.	Insecticial activity	[129]
Destruxin A_4_ chlorohydrin (**146**)	Unidentified fungus OS-F68576	Induction of erythropoietin gene expression	[130]
Destruxin A_5_ (**147**)	*Aschersonis* sp.	Insecticial activity	[129]
Destruxin B (**148**)	Entomopathogenic fungus *Metarhizium anisopliae*	Insecticidal activity	[127]
	-	Inhibitory on *Helicobacter pylori*	[131]
	Entomopathogenic fungus *Metarhizium anisopliae*	-	[126]
	Insect pathogenic fungus *Ophiocordyceps coccidiicola*	Antitrypanosomal activity on *Trypanosoma brucei* with an IC_50_ value of 0.16 μg/mL	[128]
[Phe^3^, *N*-MeVal^5^] Destruxin B (**149**)	*Beauveria felina*	-	[132]
Destruxin B_1_ (**150**)	Entomopathogenic fungus *Metarhizium anisopliae*	-	[126]
Destruxin B_2_ (**151**)	Entomopathogenic fungus *Metarhizium anisopliae*	-	[126]
	*Alternaria brassicae*	-	[133]
Dextruxin B_4_ = Homodestruxin B (**152**)	*Alternaria brassice*	-	[121]
	*Aschersonis* sp.	-	[129]
Destruxin C (**153**)	Entomopathogenic fungus *Metarhizium anisopliae*	Insecticidal activity	[120,126]
Destruxin C_1_ (**154**)	*Metarhizium brunneum*	-	[134]
Destruxin C_2_ (**155**)	Entomopathogenic fungus *Metarhizium anisopliae*	-	[126]
Destruxin D (**156**)	Entomopathogenic fungus *Metarhizium anisopliae*	Insecticidal activity	[120,126]
Destruxin D_1_ (**157**)	Entomopathogenic fungus *Metarhizium anisopliae*	-	[126]
Destruxin D_2_ (**158**)	Entomopathogenic fungus *Metarhizium anisopliae*	-	[126]
Destruxin E (**159**)	Entomopathogenic fungus *Metarhizium anisopliae*	Insecticidal activity	[126]
Destruxin E chlorohydrin (**160**)	*Beauveria felina* EN-135	-	[125]
	Entomopathogenic fungus *Metarhizium anisopliae*	Insecticidal activity	[127]
	Insect pathogenic fungus *Ophiocordyceps coccidiicola*	Antitrypanosomal activity on *Trypanosoma brucei* with an IC_50_ value of 0.061 μg/mL	[128]
[*β*-Me-Pro] Destruxin E chlorohydrin (**161**)	Marine-derived fungus *Beauveria felina*	-	[135]
	*Beauveria felina* EN-135	-	[125]
Destruxin E_1_ (**162**)	Entomopathogenic fungus *Metarhizium anisopliae*	-	[126]
Destruxin E_2_ (**163**)	Entomopathogenic fungus *Metarhizium anisopliae*	Insecticidal activity	[127]
Destruxin E_2_ chlorohydrin (**164**)	*Metarrhzium anisopliae*	Weak suppressive activity on the production of hepatitis B virus antigen	[136]
Destruxin Ed (**165**)	*Metarhizium anisopliae*	Insecticidal activity	[119]
Destruxin Ed_1_ (**166**)	Entomopathogenic fungus *Metarhizium anisopliae*	Insecticidal activity	[137]
Destruxin Ed_2_ (**167**)	*Metarhizium brunneum*	-	[134]
Destruxin F (**168**)	Entomopathogenic fungus *Metarhizium anisopliae*	Insecticidal activity	[119]
Destruxin G (**169**)	*Metarhizium brunneum*	-	[134]
Destruxin G_1_ (**170**)	*Metarhizium brunneum*	-	[134]
Emericellamide A (**171**)	*Aspergillus nidulans*	-	[138]
	Marine-derived fungus *Emericella* sp. From the surface of a green alga of the genus *Hamlima*	Antibacterial activity	[139]
Emericellamide B (**172**)	Marine-derived fungus *Emericella* sp. from the surface of a green alga of the genus *Hamlima*	Antibacterial activity	[139]
Emericellamide C (**173**)	*Aspergillus nidulans*	-	[138]
Emericellamide D (**174**)	*Aspergillus nidulans*	-	[138]
Emericellamide E (**175**)	*Aspergillus nidulans*	-	[138]
Emericellamide F (**176**)	*Aspergillus nidulans*	-	[138]
Enniatin A (**177**)	*Fusarium acuminatum*	-	[140]
	Endophytic fungus *Fusarium tricinctum* isolated from the fruits of *Hordeum sativm*	Insecticidal activity	[141]
	*Fusarium tricinctum*	Inducing an increase in the mitochondrial respiration	[142]
	-	Cytotoxicity on Caco-2 cells, Hep-G2 and HT-29	[143]
	-	Cytotoxicity in human hepatocarcinoma cell line HepG2	[144]
Enniatin A_1_ (**178**)	*Fusarium tricinctum*	Inducing an increase in the mitochondrial respiration	[142]
	-	Cytotoxicity on Caco-2 cells, Hep-G2 and HT-29	[143]
	Endophytic fungus *Fusarium tricinctum* isolated from the fruits of *Hordeum sativm*	Insecticidal activity	[141]
Enniatin A_2_ (**179**)	*Fusarium avenaceum* DAOM 196490	Cytotoxicity on Caco-2 cells, Hep-G2 and HT-29	[143,145]
Enniatin B (**180**)	*Acremonium* sp. BCC 28424	Antimalaria, antituberculosis and cytotoxic activities	[85]
	Endophytic fungus *Fusarium* sp. strain F31 from the needles of *Pinus sylvestris*	Inhibition on *Botrytis cinerea* spore germination	[146]
	*Fusarium tricinctum*	Inducing an increase in the mitochondrial respiration	[142]
	-	Cytotoxicity on Caco-2 cells, Hep-G2 and HT-29	[143]
	-	Cytotoxicity in human hepatocarcinoma cell line HepG2	[144]
	*Halosarpheia* sp. strain 732	-	[147]
	*Fusarium acuminatum*	-	[140]
	Entomogenous fungus *Fusarium proliferatum* from the cadaver of an unidentified insect collected in Tibet	-	[86]
	Endophytic fungus *Fusarium tricinctum* isolated from the fruits of *Hordeum sativm*	Insecticidal activity	[141]
	*Verticillium hemipterigenum*	-	[148]
Enniatin B_1_ (**181**)	*Fusarium acuminatum*	-	[140]
	Endophytic fungus *Fusarium* sp. strain F31 from the needles of *Pinus sylvestris*	Inhibition on *Botrytis cinerea* spore germination	[146]
	-	Cytotoxicity on Caco-2 cells, Hep-G2 and HT-29	[143]
	*Fusarium tricinctum*	Inducing an increase in the mitochondrial respiration	[142]
	Endophytic fungus *Fusarium tricinctum* isolated from the fruits of *Hordeum sativm*	Insecticidal activity	[141]
Enniatin B_2_ (**182**)	*Fusarium acuminatum*	-	[140]
	Endophytic fungus *Fusarium* sp. strain F31 from the needles of *Pinus sylvestris*	Inhibition on *Botrytis cinerea* spore germination	[146]
	Endophytic fungus *Fusarium tricinctum* isolated from the fruits of *Hordeum sativm*	Insecticidal activity	[141]
Enniatin B_3_ (**183**)	*Fusarium acuminatum*	-	[140]
Enniatin B_4_ = Enniatin D(**184**)	*Fusarium acuminatum*	-	[140]
	*Fusarium* sp. FO-1305	ACAT inhibition	[91]
	*Fusarium tricinctum*	Inducing an increase in the mitochondrial respiration	[142]
	Endophytic fungus *Fusarium* sp. strain F31 from the needles of *Pinus sylvestris*	Inhibition on *Botrytis cinerea* spore germination	[146]
	-	Cytotoxicity on Caco-2 cells, Hep-G2 and HT-29	[143]
	*Halosarpheia* sp. strain 732	-	[147]
	*Verticillium hemipterigenum*	-	[148]
Enniatin C (**185**)	*Verticillium hemipterigenum*	-	[148]
Enniatin E_1_ (**186**)	*Fusarium* sp. FO-1305	ACAT inhibition	[91]
Enniatin E_2_ (**187**)	*Fusarium* sp. FO-1305	ACAT inhibition	[91]
Enniatin F (**188**)	*Fusarium* sp. FO-1305	ACAT inhibition	[91]
Enniatin G (**189**)	*Halosarpheia* sp. strain 732	Cyctotoxic activity on Heps 7402, with an ED_50_ of 12 μg/mL	[147]
	*Verticillium hemipterigenum*	-	[148]
Enniatin H (**190**)	*Fusarium oxysporum* KFCC 11363P	Cytotoxic activity	[93]
	*Verticillium hemipterigenum*	-	[148]
Enniatin I (**191**)	*Fusarium oxysporum* KFCC 11363P	Cytotoxic activity	[93]
	*Verticillium hemipterigenum*	-	[148]
	Entomogenous fungus *Fusarium proliferatum* from the cadaver of an unidentified insect collected in Tibet	-	[86]
Enniatin J_1_ (**192**)	Endophytic fungus *Fusarium* sp. strain F31 from the needles of *Pinus sylvestris*	Inhibition on *Botrytis cinerea* spore germination	[146]
	*Fusarium solani*	Antibacterial effects on pathogenic and lactic acid bacteria	[149]
	*Fusarium tricinctum*	Inducing an increase in the mitochondrial respiration	[142]
Enniatin J_2_ (**193**)	Endophytic fungus *Fusarium* sp. strain F31 from the needles of *Pinus sylvestris*	Inhibition on *Botrytis cinerea* spore germination	[146]
Enniatin J_3_ (**194**)	*Fusarium solani*	Antibacterial effects on pathogenic and lactic acid bacteria	[149]
	Endophytic fungus *Fusarium* sp. strain F31 from the needles of *Pinus sylvestris*	Inhibition on *Botrytis cinerea* spore germination	[146]
	-	Cytotoxicity on Caco-2 cells, Hep-G2 and HT-29	[143]
Enniatin K_1_ (**195**)	Endophytic fungus *Fusarium* sp. strain F31 from the needles of *Pinus sylvestris*	Inhibition on *Botrytis cinerea* spore germination	[146]
	Entomogenous fungus *Fusarium proliferatum* from the cadaver of an unidentified insect collected in Tibet	-	[86]
Enniatin L (**196**)	Entomogenous fungus *Fusarium proliferatum* from the cadaver of an unidentified insect collected in Tibet	Antimalarial, antituberculous and cytotoxic activities	[86]
	*Acremonium* sp. BCC 2629	-	[150]
Enniatin M_1_ (**197**)	*Acremonium* sp. BCC 2629	Antimalarial, antituberculous and cytotoxic activities	[150]
Enniatin M_2_ (**198**)	*Acremonium* sp. BCC 26299	Antimalarial, antituberculous and cytotoxic activities	[150]
Enniatin MK1688 (**199**)	*Fusarium oxysporum* KFCC 11363P	Cytotoxic activity	[93]
	*Fusarium oxysporum* FB1501	Cytotoxic effects on several adenocarcinoma cell lines	[151]
	*Fusarium oxysporum*	-	[152]
	*Verticillium hemipterigenum*	-	[148]
Enniatin N (**200**)	*Acremonium* sp. BCC 2629	Antimalarial, antituberculous and cytotoxic activities	[150]
Enniatin O_1_ (**201**)	*Verticillium hemipterigenum* BCC 1449	Antimalarial, antituberculous and cytotoxic activities	[153]
Enniatin O_2_ (**202**)	*Verticillium hemipterigenum* BCC 1449	Antimalarial, antituberculous and cytotoxic activities	[153]
Enniatin O_3_ (**203**)	*Verticillium hemipterigenum* BCC 1449	Antimalarial, antituberculous and cytotoxic activities	[153]
Enniatin P_1_ (**204**)	*Fusarium* sp. VI 03441	-	[154]
Enniatin P_2_ (**205**)	*Fusarium* sp. VI 03441	-	[154]
Enniatin Q (**206**)	Endophytic fungus *Fusarium tricinctum* isolated from the fruits of *Hordeum sativm*	Insecticidal activity	[141]
Enniatin R (**207**)	Entomogenous fungus *Fusarium proliferatum* from the cadaver of an unidentified insect collected in Tibet	-	[86]
Enniatin S (**208**)	Entomogenous fungus *Fusarium proliferatum* from the cadaver of an unidentified insect collected in Tibet	-	[86]
Enniatin T (**209**)	Entomogenous fungus *Fusarium proliferatum* from the cadaver of an unidentified insect collected in Tibet	-	[86]
Enniatin U (**210**)	Entomogenous fungus *Fusarium proliferatum* from the cadaver of an unidentified insect collected in Tibet	-	[86]
Enniatin V (**211**)	Entomogenous fungus *Fusarium proliferatum* from the cadaver of an unidentified insect collected in Tibet	-	[86]
Exumolide A (**212**)	Marine-derived fungus *Scytalidium* sp. obtained from decying plant material in the Exuma Islands, Bahamas	Antimicroalgal activity	[155]
Exumolide B (**213**)	Marine-derived fungus *Scytalidium* sp. obtained from decying plant material in the Exuma Islands, Bahamas	Antimicroalgal activity	[155]
Guangomide A (**214**)	Endophytic fungus *Acremonium* sp. PSU-MA70 from a mangrove *Rhizophora apiculata*	-	[156]
	*Trichothecium* sp. MSX 51320	-	[105]
	Unidentified sponge-derived fungus	Weak antibacterial activity on *Staphylococcus epidermids* and *Enterococcus durans*	[106]
Guangomide B (**215**)	Endophytic fungus *Acremonium* sp. PSU-MA70 from a mangrove *Rhizophora apiculata*	-	[156]
	Unidentified sponge-derived fungus	Weak antibacterial activity on *Staphylococcus epidermids* and *Enterococcus durans*	[106]
Hirsutatin A (**216**)	Insect pathogenic fungus *Hirsutella nivea* BCC 2594 from a Homoptera leaf-hoppper	-	[157]
Hirsutatin B (**217**)	Insect pathogenic fungus *Hirsutella nivea* BCC 2594 from a Homoptera leaf-hoppper	Antimalarial activity on *Plasmodium falciparum* K1 with an IC_50_ value of 5.8 μg/mL	[157]
Hirsutellide A (**218**)	Entomopathogenic fungus *Hirsutella kobayasii*	Antimycobacterial activity; antimalarial activity on *Plasmodium falciparum*	[94]
Homodestcardin (**219**)	Unidentified fungus 001314c from *Ianthella* sp.	-	[106]
Hydroxydestruxin B (**220**)	*Alternaria brassicae*	Phytotoxic activity	[158]
Hydroxyhomodestruxin B (**221**)	*Alternaria brassicae*	Phytotoxic activity	[158]
IB-01212 (**222**)	*Clonostachys* sp. ESNA-A009	Cytotoxic activity	[159]
	*Clonostachys* sp.	Antitumoral activity	[160]
Isaridin A (**223**)	*Beauveria* sp. Lr89	-	[161]
	*Beauveria felina* EN-135	-	[99]
	*Isaria* sp. from soil	-	[162]
Isaridin B (**224**)	*Beauveria felina* EN-135	-	[99]
	*Isaria* sp. from soil	-	[162]
Isaridin C_1_ (**225**)	*Isaria* sp. from soil	-	[98]
Isaridin C_2_ (**226**)	*Isaria* sp. from soil	-	[98]
	*Beauveria felina*	-	[123]
Isaridin C_1_ (**225**)/C_2_ (**226**) = Isarfelin A	*Isaria felina*	Antifungal and insecticidal activities	[95]
Isaridin D (**227**)	*Isaria* sp. from soil	-	[98]
Isaridin E = Isarfelin B (**228**)	*Isaria felina*	Antifungal and insecticidal activities	[95]
	*Isaria felina* KMM 4639	-	[163]
	*Beauveria felina* EN-135	-	[99]
	*Beauveria felina*	-	[123]
Isaridin F (**229**)	*Beauveria felina*	-	[123]
Isaridin G (**230**)	*Beauveria felina* EN-135	-	[99]
Isariin A = Isariin (**231**)	*Isaria felina*	Insecticidal activity	[98]
Isariin B (**232**)	*Isaria felina*	Insecticidal activity	[97]
Isariin C (**233**)	*Isaria felina*	Insecticidal activity	[97]
Isariin C_2_ (**234**)	*Isaria felina*	Insecticidal activity	[98]
Isariin D (**235**)	*Isaria felina*	Insecticidal activity	[97]
Isariin E (**236**)	*Isaria felina*	Insecticidal activity	[98]
Isariin F_2_ (**237**)	*Isaria felina*	Insecticidal activity	[98]
Isariin G_1_ (**238**)	*Isaria felina*	Insecticidal activity	[98]
Isariin G_2_ (**239**)	*Isaria felina*	Insecticidal activity	[98]
Isoisariin B (**240**)	*Isaria felina* KMM 4639	-	[163]
	*Beauveria felina*	Insecticidal activity	[96]
Isoisariin D (**241**)	*Beauveria felina* EN-135	Brine-shrimp lethality activity	[125]
Nodupetide (**242**)	*Nodulisporium* sp. IFB-A163 residing in the gut of insect *Riptortus pedestris*	Insecticidal and antimicrobial activities	[100]
Oryzamide A (**243**)	Marine-derived fungus *Nigrospora oyzae* from the sponge *Phakellia fusca*	-	[164]
Oryzamide B (**244**)	Marine-derived fungus *Nigrospora oyzae* from the sponge *Phakellia fusca*	-	[164]
Oryzamide C (**245**)	Marine-derived fungus *Nigrospora oyzae* from the sponge *Phakellia fusca*	-	[164]
Oryzamide D (**246**)	Marine-derived fungus *Nigrospora oyzae* from the sponge *Phakellia fusca*	-	[164]
Oryzamide E (**247**)	Marine-derived fungus *Nigrospora oyzae* from the sponge *Phakellia fusca*	-	[164]
Paecilodepsipeptide A = Gliotide (**248**)	Marine-derived fungus *Gliocladium* sp. from the alga *Durvillaea antarctica*	-	[101]
	Insect pathogenic fungus *Paecilomyces cinnamomeus* BCC 9616	Antimalarial and cytotoxic activities	[102]
Pseudodestruxin A (**249**)	Coprophilous fungus *Nigrosabulum globosum*	Antibacterial activity	[103]
Pseudodestruxin B (**250**)	Coprophilous fungus *Nigrosabulum globosum*	Antibacterial activity	[103]
Pseudodestruxin C (**251**)	Marine-derived fungus *Beauveria felina*	-	[135]
Pullularin A (**252**)	*Pullularia* sp. BCC 8613	Antimalarial, antiviral and cytotoxic activities	[165]
	*Bionectria ochroleuca*	Cytotoxic activity on L5178Y cell line	[166]
Pullularin B (**253**)	*Pullularia* sp. BCC 8613	-	[165]
Pullularin C (**254**)	*Pullularia* sp. BCC 8613	-	[165]
	*Verticillium* F04W2166	Inhibitory activity on proteasome; Cytotoxic activity on human colon cell line HT-29 and human breast cancer cell line MDA-MB-231	[167]
	-	Cytotoxic acvitiy on human PC-3 cells	[168]
	*Bionectria ochroleuca*	Cytotoxic activity on L5178Y cell line	[166]
Pullularin D (**255**)	*Pullularia* sp. BCC 8613	-	[165]
Pullularin E (**256**)	Endophytic fungus *Bionecteria ochroleuca* from the mangrove plant *Sonneratia caseolaris*	Cytotoxic activity on L5178Y cell line	[166]
Roseocardin (**257**)	*Beauveria felina*	Antibacterial activity	[123]
	*Trichothecium roseum* TT103	Positive inotropic effect on rat heart muscles	[169]
Roseotoxin A (**258**)	*Trichothecium roseum*	-	[170]
Roseotoxin B (**259**)	*Beauveria felina*	-	[123]
	*Beauveria felina* EN-135	Lethality against brine shrimp with an LD_50_ value of 0.73 μM	[125]
	*Trichothecium roseum* TT1031	-	[169]
	*Trichothecium roseum*	-	[171]
	*Trichothecium roseum*	Phtotoxic activity	[172]
Roseotoxin C (**260**)	*Trichothecium roseum*	-	[170]
Scopularide A (**261**)	Marine sponge-derived *Scopulariopsis brevicaulis* from *Tethya aurantium*	Cytotoxic activity	[173]
Scopularide B (**262**)	Marine sponge-derived *Scopulariopsis brevicaulis* from *Tethya aurantium*	Cytotoxic activity	[173]
Spicellamide A (**263**)	Marine-derived fungus *Spicellum roseum* from the sponge *Ectyplasia perox*	Cytotoxic activity	[174]
Spicellamide B (**264**)	Marine-derived fungus *Spicellum roseum* from the sponge *Ectyplasia perox*	Cytotoxic activity	[174]
Sporidesmolide I (**265**)	*Pithomyces chartarum*	-	[175]
Sporidesmolide II (**266**)	*Pithomyces chartarum*	-	[175]
Sporidesmolide III (**267**)	*Pithomyces chartarum*	-	[175]
Sporidesmolide IV (**268**)	*Pithomyces chartarum*	-	[176]
Sporidesmolide V (**269**)	*Pithomyces chartarum*	-	[177]
T987A (**270**)	*Cladobotryum* sp.	Cytotoxic activity	[178]
T987B (**271**)	*Cladobotryum* sp.	Cytotoxic activity	[178]
Trichodepsipeptide A (**272**)	*Trichothecium* sp. MSX 51320	-	[105]
Trichodepsipeptide B (**273**)	*Trichothecium* sp. MSX 51320	-	[105]
Trichomide A (**274**)	*Trichothecium roseum*	Immunosuppressive activity	[107]
Trichomide B (**275**)	*Trichothecium roseum*	Immunosuppressive activity	[107]
Verticilide B_1_ (**276**)	*Verticillium* sp. FKI-2679 from soil	Inhibition of ACAT1 and ACAT2	[179]

Note. ACAT, acyl-CoA: cholesterol acyltransferase; ED_50_, median effective dose. IC_50_, median inhibitory concentration. LD_50_, median lethal dose.

**Table 5:** Fungal cyclic heptadepsipeptides and their biological activities.

Name	Fungus and Its Origin	Biological Activity	References
Cordycommunin (**277**)	*Ophiocordyceps communis* BCC16475	Antimycobacterial activity; Cytotoxic activity	[180]
Fusaripeptide A (**278**)	Endophytic fungus *Fusarium* sp. from *Mentha longifolia*	Antifungal, anti-malarial and cytotoxic activities	[181]
HUN-7293 (**279**)	Unidentified fungus	Inhibition of inducible cell adhesion molecule expression	[186]
Simplicilliumtide J (**280**)	Deep-sea derived fungus *Simplicillium obclavatum*	Antifungal and antiviral activities	[182]
Simplicilliumtide K (**281**)	Deep-sea derived fungus *Simplicillium obclavatum*	-	[182]
Simplicilliumtide L (**282**)	Deep-sea derived fungus *Simplicillium obclavatum*	-	[182]
Verlamelin A (**283**)	Entomopathogenic fungus *Lecanicillium* sp.	Antifungal activity	[183]
	Deep-sea derived fungus *Simplicillium obclavatum*	Antifungal and antiviral activities	[182]
Verlamelin B (**284**)	Entomopathogenic fungus *Lecanicillium* sp.	Antifungal activity	[183]
	Deep-sea derived fungus Simplicillium obclavatum	Antifungal and antiviral activities	[182]
W493 A (**285**)	Endophytic fungus *Fusarium* sp. from *Ceriops tagal*	Antifungal activity	[185]
W493 B (**286**)	Endophytic fungus *Fusarium* sp. from *Ceriops tagal*	Antifungal activity	[185]
	*Fusarium* sp. CANU-HA23	Antifungal activity	[25]
W493 C (**287**)	Endophytic fungus *Fusarium* sp. from *Ceriops tagal*	-	[184]
W493 D (**288**)	Endophytic fungus *Fusarium* sp. from *Ceriops tagal*	-	[184]

**Table 6:** Fungal cyclic octadepsipeptides and their biological activities.

Name	Fungus and Its Origin	Biological Activity	References
Bassianolide (**289**)	*Beauveria bassiana*; *Lecanicilium* sp. (formerly *Verticillium lecanii*)	Insecticidal, cytotoxic and anthelmintic acitivities	[187,188]
	*Xylaria* sp. BCC1067	-	[189]
BZR-cotoxin IV (**290**)	Plant pathogenic fungus *Bipolaris zeicola*	-	[197]
	Plant endopytic fungus *Bipolaris sorokiniana* LK12	Moderate anti-lipid peroxidation and urease activities	[198]
Glomosporin (**291**)	*Glomospora* sp. BAUA 2825	Antifungal activity	[199,200]
Halobacillin (**292**)	*Trichoderma asperellum*	Antibacterial activity	[201]
PF1022A (**293**)	Endophytic fungus *Rosellina* sp. PF1022	Anthelmintic activity on *Ascaridia galli* in chicken	[191]
	*Mycelia sterilia* PF1022	Anthelmintic activity	[192]
PF1022B (**294**)	*Mycelia sterilia* PF1022	Anthelmintic activity	[192]
PF1022C (**295**)	*Mycelia sterilia* PF1022	Anthelmintic activity	[192]
PF1022D (**296**)	*Mycelia sterilia* PF1022	Anthelmintic activity	[192]
PF1022E (**297**)	*Mycelia sterilia* PF1022	Anthelmintic activity	[192]
PF1022F (**298**)	*Mycelia sterilia* PF1022	Anthelmintic activity	[192]
	*Trichoderma asperellum*	Antibacterial activity	[201]
PF1022G (**299**)	*Mycelia sterilia* PF1022	Anthelmintic activity	[192]
PF1022H (**300**)	*Mycelia sterilia* PF1022	Anthelmintic activity	[192]
Phaeofungin (**301**)	Endophytic fungus *Phaeosphaeria* sp. from *Sedum* sp.	Causing ATP release in wild-type *Candida albicans* strains; Modest antifungal activity	[196]
Verticilide = Verticilide A1 (**302**)	*Verticillium* sp. FKI-1033 from soil	Selectively binding to the insect ryanodine receptor	[202]
	*Verticillium* sp. FKI-2679 from soil	ACAT inhibition	[179]
Verticilide A2 (**303**)	*Verticillium* sp. FKI-2679 from soil	ACAT inhibition	[179]
Verticilide A3 (**304**)	*Verticillium* sp. FKI-2679 from soil	ACAT inhibition	[179]

Note. Abbreviations: ACAT, acyl-CoA: cholesterol acyltransferase.

**Table 7:** Fungal cyclic nonadepsipeptides and their biological activities.

Name	Fungus and Its Origin	Biological Activity	References
Aureobasidin A (**305**)	*Aureobasidium pullulans* from a leaf collected at Tsushima of Japan	Antifungal activity; Inhibitory activity on *Candida* planktonic and biofilm cells	[203,211,212]
Aureobasidin B (**306**)	*Aureobasidium pullulans* from a leaf collected at Tsushima of Japan	Antifungal activity	[204,213]
Aureobasidin C (**307**)	*Aureobasidium pullulans* from a leaf collected at Tsushima of Japan	Antifungal activity	[204,213]
Aureobasidin D (**308**)	*Aureobasidium pullulans* from a leaf collected at Tsushima of Japan	Antifungal activity	[204,213]
Aureobasidin E (**309**)	*Aureobasidium pullulans* from a leaf collected at Tsushima of Japan	Antifungal activity	[204,213]
Aureobasidin F (**310**)	*Aureobasidium pullulans* from a leaf collected at Tsushima of Japan	Antifungal activity	[204,213]
Aureobasidin G (**311**)	*Aureobasidium pullulans* from a leaf collected at Tsushima of Japan	Antifungal activity	[204,213]
Aureobasidin H (**312**)	*Aureobasidium pullulans* from a leaf collected at Tsushima of Japan	Antifungal activity	[204,213]
Aureobasidin I (**313**)	*Aureobasidium pullulans* from a leaf collected at Tsushima of Japan	Antifungal activity	[204,213]
Aureobasidin J (**314**)	*Aureobasidium pullulans* from a leaf collected at Tsushima of Japan	Antifungal activity	[204,213]
Aureobasidin K (**315**)	*Aureobasidium pullulans* from a leaf collected at Tsushima of Japan	Antifungal activity	[204,213]
Aureobasidin L (**316**)	*Aureobasidium pullulans* from a leaf collected at Tsushima of Japan	Antifungal activity	[204,213]
Aureobasidin M (**317**)	*Aureobasidium pullulans* from a leaf collected at Tsushima of Japan	Antifungal activity	[204,213]
Aureobasidin N (**318**)	*Aureobasidium pullulans* from a leaf collected at Tsushima of Japan	Antifungal activity	[204,213]
Aureobasidin O (**319**)	*Aureobasidium pullulans* from a leaf collected at Tsushima of Japan	Antifungal activity	[204,213]
Aureobasidin P (**320**)	*Aureobasidium pullulans* from a leaf collected at Tsushima of Japan	Antifungal activity	[204,213]
Aureobasidin Q (**321**)	*Aureobasidium pullulans* from a leaf collected at Tsushima of Japan	Antifungal activity	[204,213]
Aureobasidin R (**322**)	*Aureobasidium pullulans* from a leaf collected at Tsushima of Japan	Antifungal activity	[204,213]
Aureobasidin S_1_ (**323**)	*Aureobasidium pullulans* from a leaf collected at Tsushima of Japan	Antifungal activity	[205]
Aureobasidin S_2a_ (**324**)	*Aureobasidium pullulans* from a leaf collected at Tsushima of Japan	Antifungal activity	[205]
Aureobasidin S_2b_ (**325**)	*Aureobasidium pullulans* from a leaf collected at Tsushima of Japan	Antifungal activity	[205]
Aureobasidin S_3_ (**326**)	*Aureobasidium pullulans* from a leaf collected at Tsushima of Japan	Antifungal activity	[205]
Aureobasidin S_4_ (**327**)	*Aureobasidium pullulans* from a leaf collected at Tsushima of Japan	Antifungal activity	[205]
Aureobasidin T_1_ (**328**)	*Aureobasidium pullulans* from a leaf collected at Tsushima of Japan	Antifungal activity	[206]
Aureobasidin T_2_ (**329**)	*Aureobasidium pullulans* from a leaf collected at Tsushima of Japan	Antifungal activity	[206]
Aureobasidin T_3_ (**330**)	*Aureobasidium pullulans* from a leaf collected at Tsushima of Japan	Antifungal activity	[206]
Aureobasidin T_4_ (**331**)	*Aureobasidium pullulans* from a leaf collected at Tsushima of Japan	Antifungal activity	[206]
Aureobasidin U_1_ (**332**)	*Aureobasidium pullulans* from a leaf collected at Tsushima of Japan	Antifungal activity	[206]
Aureobasidin U_2_ (**333**)	*Aureobasidium pullulans* from a leaf collected at Tsushima of Japan	Antifungal activity	[206]
BZR-cotoxin I (**334**)	Plant pathogenic fungus *Bipolaris zeicola*	-	[208]
	Plant endopytic fungus *Bipolaris sorokiniana* LK12	Moderate anti-lipid peroxidation and uease activities	[198]
BZR-cotoxin II (**335**)	Plant pathogenic fungus *Bipolaris zeicola*	-	[214]
BZR-cotoxin III (**336**)	Plant pathogenic fungus *Bipolaris zeicola*	-	[215]
Phomafungin (**337**)	*Phoma* sp.	Antifungal activity	[216]
Pleofungin A (**338**)	*Phoma* sp. SANK 13899 from a soil sample collected at Tokyo of Japan	Inhibitory activity on inositol phosphorylceramide synthase	[209,210]
Pleofungin B (**339**)	*Phoma* sp. SANK 13899 from a soil sample collected at Tokyo of Japan	Inhibitory activity on inositol phosphorylceramide synthase	[209,210]
Pleofungin C (**340**)	*Phoma* sp. SANK 13899 from a soil sample collected at Tokyo of Japan	Inhibitory activity on inositol phosphorylceramide synthase	[209,210]
Pleofungin D (**341**)	*Phoma* sp. SANK 13899 from a soil sample collected at Tokyo of Japan	Inhibitory activity on inositol phosphorylceramide synthase	[209,210]

**Table 8:** Fungal cyclic decadepsipeptides and their biological activities.

Name	Fungus and Its Origin	Biological Activity	References
Clavariopsin A (**342**)	Aquatic hyphomycetes *Clavariopsis aquatic*	Antifungal activity	[217,218]
Clavariopsin B (**343**)	Aquatic hyphomycetes *Clavariopsis aquatic*	Antifungal activity	[217,218]
Eujavanicin A (**344**)	*Eupenicillium javanicum*	Antifungal activity	[221]
Pleosporin A (**345**)	Unidentified elephant dung fungus of the family Pleosporaceae	Antimalarial activity	[222]
Sch 217048 (**346**)	Unidentified fungus	Neurokinin antagonist activity	[223]
	-	Inhition on tachykinin receptor	[219]
	Unidentified elephant dung fungus of the family Pleosporaceae	Antimalarial activity on *Plasmodium falciparum* K1	[222]
	Freshwater fungus *Clohesyomyces aquaticus*	-	[220]
Sch 218157 (**347**)	Unidentified elephant dung fungus of the family Pleosporaceae	Antimalarial activity on *Plasmodium falciparum* K1	[222]
Sch 378161 (**348**)	Unidentified fungus	Inhition on tachykinin receptor	[219]
	Freshwater fungus *Clohesyomyces aquaticus*	-	[220]
Sch 378167 (**349**)	Unidentified fungus	Inhition on tachykinin receptor	[219]
Sch 378199 (**350**)	Unidentified fungus	Inhition on tachykinin receptor	[219]
